# Evaluating the potential of RNA interference for control of striped cucumber beetle, *Acalymma vittatum* (Fabricius) (Coleoptera: Chrysomelidae)

**DOI:** 10.1002/ps.70481

**Published:** 2025-12-25

**Authors:** Emine Kaplanoglu, Félix Longpré, Cam Donly

**Affiliations:** ^1^ London Research and Development Centre, Agriculture and Agri‐Food Canada London Canada

**Keywords:** Coleoptera, dsRNase, injected dsRNA, oral dsRNA, RNA interference

## Abstract

**BACKGROUND:**

The striped cucumber beetle (SCB) is a serious pest of cucurbit crops, causing damage both by feeding on plants and by vectoring plant diseases. Cultural, biological and chemical methods are currently used for its management, however, RNA interference (RNAi) as a potential control strategy, has not yet been evaluated.

**RESULTS:**

Injecting dsRNA into the hemocoel of adult SCB resulted in significant gene knockdown and mortality for all seven genes tested (*v‐ATPaseA*, *rpt3*, *rop*, *α‐snap*, *srp54k*, *β‐actin* and *α‐tubulin*). However, oral delivery of the three dsRNAs found to be most lethal using injections, targeting *β‐actin*, *α‐snap* and *rpt3,* led to less efficient gene knockdown and mortality than injections. *In silico* analysis of the SCB transcriptome revealed the presence of five dsRNA‐degrading nucleases, of which *dsRNase5* had the highest expression in the gut. However, double knockdown of *dsRNase5* and the *rpt3* gene did not improve oral RNAi. Comparing dsRNA stability in digestive fluid and hemolymph of SCB and Colorado potato beetle (CPB) revealed differences in dsRNA‐degrading nuclease activity. Furthermore, oral RNAi of the *β‐actin* gene in CPB adults resulted in 100% mortality, whereas mortality was only 33.3% in SCB.

**CONCLUSION:**

Although SCB has a robust RNAi response to injected dsRNA, oral RNAi works less efficiently. Knockdown of the most highly expressed dsRNase gene in the SCB gut did not enhance oral RNAi. This suggests nucleases may not be the main reason for the reduced oral RNAi efficiency in SCB and other factors are likely to be involved. © 2025 His Majesty the King in Right of Canada. *Pest Management Science* published by John Wiley & Sons Ltd on behalf of Society of Chemical Industry. Reproduced with the permission of the Minister of Agriculture and Agri‐Food.

## Graphical abstract

SCB has a robust RNAi response to injected dsRNA, but oral RNAi is less efficient. Nucleases do not appear to be responsible for the discrepancy, suggesting other factors are involved.

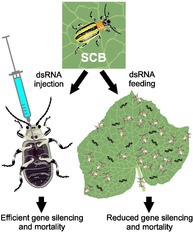

## 1 INTRODUCTION

Striped cucumber beetle (SCB, *Acalymma vittatum* (Fabricius), Coleoptera: Chrysomelidae) is a serious pest of cucurbit crops including cucumber, squash, melon and pumpkin. It is mainly localized in Eastern North America, ranging from Southeast Canada to Mexico, and causes serious crop damage by feeding on plant roots and leaves/fruits as larvae and adults, respectively.^1^ Additionally, the insect acts as a vector for plant pathogens, such as *Erwinia tracheiphila* ^2^ (Smith), *Fusarium oxysporum* (Schltdl)^3^ and squash mosaic virus.^4^ Traditionally, SCB management relies on cultural, biological and conventional chemical insecticide controls.^5^, ^6^, ^7^ However, existing control strategies may have limited efficacy in some cases, or be susceptible to the development of insecticide resistance, in others. As a more sustainable and insect‐specific control strategy, RNA interference (RNAi) can be considered.

RNAi utilizes RNA molecules, such as long double‐stranded RNA (dsRNA)^8^ to selectively eliminate or reduce gene expression in eukaryotic organisms. For pest control, exogenous dsRNAs targeting specific insect genes are introduced to insects through feeding.^9^, ^10^ The ingested dsRNA is taken up by gut cells through clathrin‐dependent endocytosis^11^, ^12^ or via dsRNA channels^13^ and activates the RNAi pathway, whereby dsRNA is processed into small interfering RNA (siRNA) molecules by the dicer enzymes.^8^ These siRNA molecules are then loaded into the RNA‐induced silencing complex (RISC), which facilitates complementary base‐pairing between siRNA and target gene messenger RNAs (mRNAs), leading to degradation of the mRNA or translational arrest.^14^

The discovery that exogenous long dsRNA molecules can activate the RNAi mechanism to knockdown gene expression has opened the possibility of using this process for pest management. For this purpose, developing transgenic plant varieties expressing insect‐specific dsRNA and spraying of dsRNA formulations onto host plants have been explored.^15^, ^16^, ^17^, ^18^, ^19^, ^20^ One of the significant milestones in using RNAi for crop protection was development of transgenic corn producing dsRNA for the *snf7* gene of Western corn rootworm (WCR), *Diabrotica virgifera virgifera* (LeConte). The corn variety provides protection against corn rootworm and is commercially available under the trademark SmartStax PRO.^21^ Likewise, a newly developed sprayable dsRNA biopesticide, (Ledprona), targeting proteasome subunit beta 5 protein has been shown to provide effective protection against Colorado potato beetle (CPB), *Leptinotarsa decemlineata* (Say).^22^

Although the above‐mentioned examples show significant potential for developing species‐specific pest control using RNAi, many studies indicate that not all insects are amenable to RNAi‐induced gene knockdown and mortality. Specifically, although coleopteran insects respond to RNAi‐based gene knockdown well,^15^, ^23^, ^24^, ^25^ lepidopteran insects, such as moths and butterflies, are often refractory to RNAi,^26^ and other insect orders show variable responses.^27^ Differences in RNAi response among insect orders are attributed to multiple factors, including abundance and activity of dsRNA‐degrading nucleases (dsRNases) in insect guts, efficiency of dsRNA uptake by gut cells via endocytosis or dsRNA channels, presence of dsRNA‐stabilizing proteins, bodily fluid pH and efficiency of dsRNA processing into siRNA, to name a few.^27^ For example, previous studies have demonstrated that in lepidopteran insects, most orally administered dsRNA is rapidly degraded by gut nucleases before cellular uptake can take place.^28^ Furthermore, even if dsRNA survives the nucleases and enters the gut cells, dsRNA molecules may become trapped in endosomes^29^, ^30^ and not be processed into siRNA–an essential step for RNAi–and this is thought to contribute to RNAi failure in lepidopteran insects. By contrast, coleopteran insects such as CPB, WCR, mustard leaf beetle, *Phaedon cochleariae* (Fabricius) and pollen beetle, *Meligethes aeneus* (Fabricius), are highly susceptible to gene knockdown through RNAi.^15^, ^16^, ^23^, ^24^ Studies demonstrate that one of the major contributing factors for this is the stability of orally delivered dsRNA, which remain intact longer in coleopteran insect guts compared with other insect orders.^30^ Additionally, efficient processing of dsRNA into siRNA is facilitated by a coleopteran‐specific protein, StaufenC, which has been shown to play a significant role in RNAi success in coleopteran insects.^31^

Given that many coleopteran pests are highly susceptible to dietary RNAi, this strategy is proposed as an alternative to conventional pesticides for coleopteran insect control.^15^, ^25^ However, a review by Willow and Veromann^32^ highlighted the high variability in response to oral RNAi in some coleopterans. For example, low efficacy of dietary RNAi has been observed in several coleopteran insects, including the pepper weevil, *Anthonomus eugenii* (Cano),^33^ the lady beetle, *Harmonia axyridis* (Pallas)^34^ and the small hive beetle, *Aethina tumida* (Murray),^35^ among others. Results from these studies underscore the importance of evaluating each insect species for RNAi efficacy. Therefore, in this study, we aimed to evaluate the effectiveness of RNAi in SCB, another coleopteran species, to determine whether RNAi could potentially provide the basis for control strategies for this pest. For this purpose, we selected seven target genes to evaluate gene knockdown and resulting mortality via dsRNA injection and feeding. Additionally, dsRNase genes in the SCB transcriptome were identified and their expression in various tissues was analyzed, followed by investigation of their possible role in RNAi efficiency in SCB. Finally, efficiency of RNAi in SCB was compared with efficiency of RNAi in CPB, which is considered to be a coleopteran model insect for RNAi studies, to reveal factors potentially contributing to the low level of oral RNAi efficiency displayed by SCB.

## 2 MATERIALS AND METHODS

### 2.1 Insect rearing

Adult SCBs were collected from squash plants at the London Research and Development Centre, London, Ontario, whereas CPBs were collected from an organic potato farm in Farnham, Quebec. Both insects were reared in screen cages at 25 °C, 60% relative humidity (RH) and 16 h:8 h, light:dark photoperiod on blue Hubbard squash plants (*Cucurbita maxima*) and potato plants (*Solanum tuberosum* var. Kennebec), respectively. For all experiments, mixed‐sex adults younger than 7 days old were used.

### 2.2 *In silico* analysis of the SCB transcriptome to select target genes

The SCB transcriptome from the USDA^36^ (TSA: *Acalymma vittatum* transcriptome shotgun assembly GHXO01000000) was imported to CLC genomics workbench v23.0.2 (Qiagen Bioinformatics, Germantown, MD, USA). Then, target and reference gene sequences from WCR or CPB were downloaded from NCBI and were used as queries in genomics workbench to find the best‐matching transcripts in the SCB transcriptome. Seven genes (*β‐actin*, *α‐snap*, *rpt3*, *rop*, *α‐tubulin*, *srp54k* and *v‐ATPaseA*), whose knockdown was shown to result in high mortality in other coleopteran insects, were selected for RNAi experiments.^15^, ^16^, ^19^, ^24^ Additionally, dsRNase gene homologs from WCR were used to identify dsRNase genes in the SCB transcriptome. PCR primers for subsequent experiments (Supporting Information, Tables S1 and S2) were designed from the gene sequences identified (Table S3).

### 2.3 *In vitro* dsRNA production

Double‐stranded RNA sequences were selected using E‐rnai web tool,^37^ and templates were PCR‐generated using SCB cDNA and gene‐specific primers. The templates were purified using a PCR clean‐up kit (Qiagen), and 5× Megascript T7 kit (Thermo Fisher Scientific, Waltham, MA, USA) was used to synthesize dsRNA following the manufacturer's instructions. For a nonspecific dsRNA control, *green fluorescent protein* gene (*gfp*) (Table S3) dsRNA was purchased from RNA Greentech LLC (Frisco, TX, USA) and was used for gene knockdown experiments.

### 2.4 RNAi using dsRNA injections in SCB

SCB adults were anaesthetized using CO_2_ on a FlyStuff Flypad (Genesee Scientific, El Cajon, CA, USA) and were injected with 0.5 μL water containing 1.0 μg dsRNA. Injections were made into the thoracic region on the ventral side of the insects using a 2.5‐μL Hamilton syringe fitted with a 33‐gauge needle (Hamilton Company, Reno, NV, USA). Insects were then provided with fresh squash leaves for 3 days, after which three individuals per biological replicate were frozen in liquid nitrogen for gene expression analysis to confirm gene knockdown. Three biological replicates were done per treatment. For mortality assessment, ≥30insects were injected per target dsRNA or *gfp* dsRNA and then were provided with fresh squash leaves. Insects were monitored daily and mortality was recorded for 10 days.

### 2.5 RNA extraction and cDNA synthesis

RNA was extracted using a Qiagen RNeasy Kit, following the manufacturer's instructions. To remove residual gDNA, RNA was treated using an Ambion Turbo RNase‐Free DNase kit (Thermo Fisher Scientific) following the manufacturer's instructions. cDNA was synthesized from 900 ng RNA using the Invitrogen Superscript III First‐Strand Supermix Kit (Thermo Fisher Scientific), following the manufacturer's instructions.

### 2.6 Quantitative reverse‐transcription (qRT)‐PCR for gene expression analysis

The qRT‐PCR reactions were performed using a SensiFAST SYBR No‐ROX Mix Kit (Meridian Bioscience, Memphis, TN, USA) following the manufacturer's recommendations. Reactions included 400 nm each of forward and reverse primers and 2.5 μL of a 1:3 dilution of cDNA template in a 10‐μL final volume. Translation elongation factor 1α (*ef1α*) and ribosomal protein l8 (*rpl8*) were used to normalize the transcript abundance of target genes in SCB, whereas ribosomal protein l8e (*l8e*) and *ef1α* were used for CPB. Samples were run in technical triplicate and all qRT‐PCR primers were tested for their amplification efficiencies to comply with MIQE guidelines for qRT‐PCR.^38^

### 2.7 RNAi using naked dsRNA feeding in SCB

For feeding bioassays, the three most lethal dsRNAs deduced from injections (*β‐actin*, *rpt3* and *α‐snap*), were selected and corresponding dsRNAs purchased from RNA Greentech LLC. dsRNA was diluted to 1.0 μg uL^−1^ in nuclease‐free water containing 0.05% (v/v) Triton X‐100 to promote leaf‐coating. The squash leaves were immersed in the dsRNA solution and were dried on a metal mesh. Adult insects were allowed to feed on the treated leaves *ad libitum*. The leaves were replaced every second day and insects were observed daily for 10 days for mortality. Gene‐knockdowns were confirmed after insects fed on treated leaves for 3 days using three biological replicates, each having three insects.

### 2.8 Production of dsRNA in *Escherichia coli* HT115 for RNAi in SCB

dsRNA targeting *β‐actin*, *rpt3* and *α‐snap* were produced in HT115 cells as described previously.^25^, ^39^, ^40^ Briefly, dsRNA fragments were cloned into pL4440 vector and transformed into HT115. Cells were induced to produce dsRNA using 1 mm isopropyl β‐d‐1‐thiogalactopyranoside and cultures were concentrated 20× in 1× phosphate‐buffered saline [PBS; pH = 7.4, 137 mm sodium chloride (NaCl), 2.7 mm potassium chloride (KCl), 10 mm disodium phosphate (Na_2_HPO_4_), 2 mm monopotassium phosphate (KH_2_PO_4_)] for feeding. HT115 transformed with empty pL4440 vector served as control. To feed SCB, squash leaves were coated with HT115 cultures and dried on a metal mesh, followed by presenting the treated leaves to SCB adults. The leaves were replaced daily, and mortality and gene knockdown were analyzed as described in previous sections. Forty insects were used per treatment.

### 2.9 Analysis of dsRNase gene expression in SCB tissues

SCB adults were dissected in Calpode's insect saline [pH = 7.2, 10.7 mm NaCl, 25.8 mm KCl, 90 mm glucose, 29 mm calcium chloride (CaCl_2_), 20 mm magnesium chloride (MgCl_2_) and 5 mm HEPES], and guts, heads and remaining carcass were isolated. Tissues from 10 insects were pooled together to form a biological replicate and a total of three replicates were done. RNA was extracted, followed by removal of gDNA. cDNA synthesis was performed as described previously using 600 ng RNA. qRT‐PCR was used to analyze relative expression of dsRNase genes in the three tissues. The dsRNase gene whose expression was the highest in SCB gut (*dsRNase5*) was selected for double knockdown experiments.

### 2.10 Expression of dsRNase genes upon exposure to dsRNA

In order to determine whether any of the dsRNase genes are specifically induced upon exposure to dsRNA, which could indicate a significant role in responding to dsRNA by degrading it, SCB adults were injected with either water or *gfp* dsRNA as described previously. After Day (D)3, total RNA was extracted, cDNA was synthesized and qRT‐PCR was performed to analyze transcript levels of dsRNase genes in whole adult bodies.

### 2.11 Double knockdown of genes in SCB

First, SCB adults were injected with 1.0 μg *dsRNase5* dsRNA as described previously. Then, squash leaves were immersed in 1.0 μg μL^−1^ *rpt3* dsRNA solution containing 0.05% (v/v) Triton X‐100. The insects were allowed to feed on the dsRNA‐coated leaves for 10 days to determine if knocking down *dsRNase5* gene levels via injections, could increase oral RNAi efficiency of the *rpt3* gene and result in higher mortality than just feeding the *rpt3* dsRNA alone. Gene knockdown was confirmed on D3 as described previously. For controls, insects were either injected and fed with *gfp* dsRNA (*gfp* + *gfp*) or injected with *dsRNase5* dsRNA and fed *gfp* dsRNA (*dsRNase5* + *gfp*).

### 2.12 RNAi using naked dsRNA feeding in CPB

In order to compare RNAi efficiency between SCB and CPB, the *β‐actin* gene was selected for dsRNA feeding bioassays for assessing gene knockdown and resulting mortality. Potato leaves were dipped in 1.0 μg μL^−1^ *β‐actin* or *gfp* dsRNA in 0.05% Triton X‐100 and dried on a metal rack. The leaves were then placed in 125‐mm‐diameter petri dishes lined with moistened Whatman paper. At least 10 CPB adults were placed in each petri dish and allowed to feed *ad libitum*. The leaves were replaced daily and mortality was monitored for 10 days. Each treatment consisted of a total of 30 beetles. Gene knockdown was confirmed at D3, and two insects per biological replicate were used for RNA extraction, cDNA synthesis and qRT‐PCR. A total of three biological replicates were performed.

### 2.13 Bodily fluid collection from SCB and CPB

In order to collect hemolymph, adult insects were cooled on ice for 15 min and then pinned on a waxy surface with their ventral side up. The hind legs were cut off, allowing hemolymph to ooze out, which was collected using a pipette tip. To collect digestive fluid, insects were placed on a FlyStuff Flypad (Genesee Scientific) and exposed to CO_2_. The insects were gently prodded with a paint brush, which caused them to expel their digestive contents from their mouths. The liquid expelled was collected using a pipette tip and is referred to as digestive fluid in this study. Samples were flash frozen in liquid nitrogen and stored at −80 °C until use.

### 2.14 Stability of dsRNA in bodily fluids of SCB and CPB

Total protein concentrations in SCB and CPB hemolymph and digestive fluid were determined using a Pierce BCA protein kit (Thermo Fisher Scientific) following the manufacturer's instructions. Then, fluid quantities containing 10 μg total protein from each bodily fluid were incubated with 1.0 μg of *gfp* dsRNA at 26 °C for 0, 30, 60, 120, 180 and 300 min. The products were then separated on a 3% agarose gel to visualize the remaining dsRNA. In a separate experiment, serial dilutions of SCB and CPB digestive fluids were incubated with dsRNA for 90 min after the digestive fluids were either pre‐treated with 100 mm EDTA or heat‐inactivated at 100 °C for 10 min, to determine whether the dsRNase activity can be inhibited. Finally, the differences in pH of the bodily fluids of the two insects also were measured using an Orion 9810BN Micro pH Electrode (Thermo Fisher Scientific).

### 2.15 Expression of *staufenC* gene in SCB and CPB

Given that *staufenC* gene is required for RNAi in coleopteran insects,^31^ its expression level was compared by qRT‐PCR between adult SCB and CPB using cDNA synthesized from RNA extracted from whole bodies as described previously. Likewise, tissue‐specific *staufenC* expression was compared between SCB head, carcass and gut tissues using cDNA prepared from each tissue as described previously.

### 2.16 Statistical analysis

The qRT‐PCR data was analyzed by the 2^−ΔΔCt^ method using Bio‐Rad cfx maestro v2.2 software, and statistical analyses were done using ANOVA followed by Tukey's honestly significant difference (HSD) tests or Student's *t*‐tests where applicable. Insect mortality was analyzed using Kaplan–Meier, followed by pairwise log‐rank tests, using the survival package in R.^41^ A *P*‐value of ≤0.05 was considered to be significantly different.

## 3 RESULTS

### 3.1 dsRNA injection into SCB results in strong RNAi response and mortality

**Figure 1:**
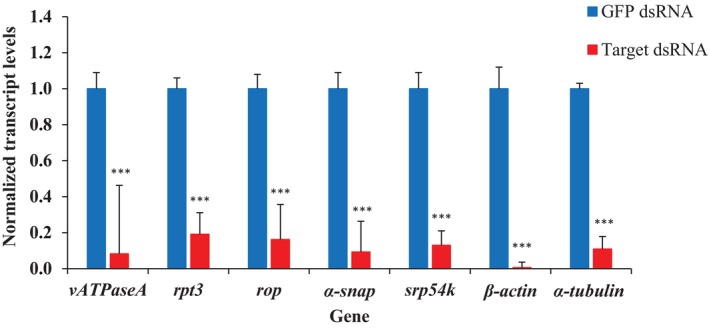
Normalized gene expression in SCB adults injected with dsRNA. Normalized transcript level was set to ‘1.0’ in *gfp* dsRNA‐injected SCB and relative transcript levels in target gene dsRNA‐injected SCB were calculated. Data are mean relative quantity ±SEM. Asterisks represent significant transcript level changes in Student's *t*‐tests (***, *P* ≤ 0.001), *n* = 3.

dsRNA injections into SCB adults resulted in significant gene knockdown for all target genes compared with *gfp* dsRNA‐injected controls. Relative transcript levels were reduced by 91.8% (12.3‐fold), 80.9% (5.3‐fold), 84.2% (6.2‐fold), 90.7% (10.7‐fold), 87.0% (7.7‐fold), 99.4% (164.4‐fold) and 89.1% (9.2‐fold) for *v‐ATPaseA*, *rpt3*, *rop*, *α‐snap*, *srp54k*, *β‐actin* and *α‐tubulin*, respectively, all of which were significant [*v‐ATPaseA* (*t* = 25.7, *P* = 1.4E^−05^), *rpt3* (*t* = 15.6, *P =* 5.7E^−04^), *rop* (*t* = 9.3, *P* = 1.0E^−03^), *α‐snap* (*t* = 21.4, *P* = 2.2E^−04^), *srp54k* (*t* = 24.7, *P* = 1.6E^−05^), *β‐actin* (*t* = 61.4, *P* = 4.2E^−07^) and *α‐tubulin* (*t* = 21.9, *P* = 2.1E^−04^)] (Fig. 1).

**Figure 2:**
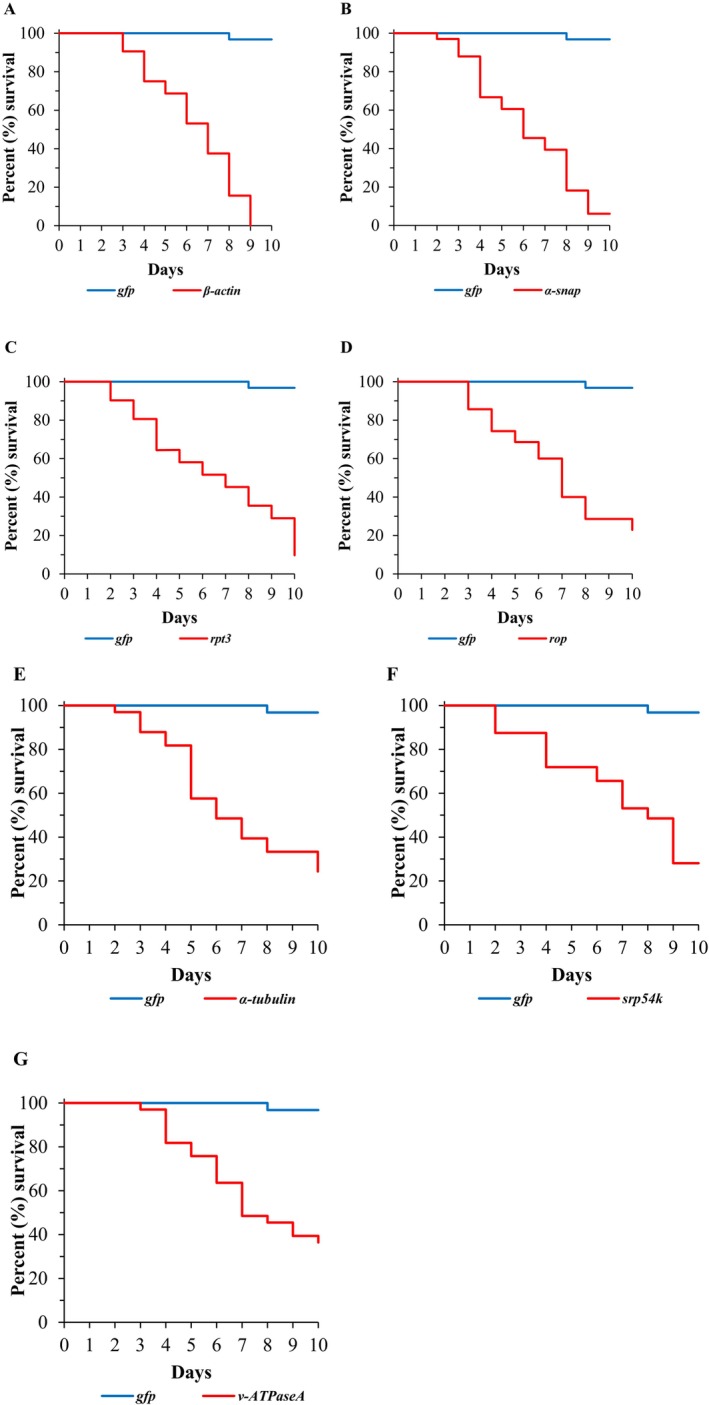
Kaplan–Meier survival curves showing the percent survival of SCB injected with dsRNA. Insects were monitored for mortality for 10 days after dsRNA injections using *gfp* or a target gene as indicated: (A) *β‐actin*, (B) *α‐snap*, (C) *rpt3*, (D) *rop*, (E) *α‐tubulin*, (F) *srp54k* and (G) *v‐ATPaseA*.

Injection of dsRNA for target genes also resulted in significant mortality in SCB: 100% for *β‐actin*, 93.9% for *α‐snap*, 90.3% for *rpt3*, 77% for *rop*, 75% for *α‐tubulin*, 71% for *srp54k* and 63.6% for *v‐ATPaseA*, whereas mortality was 3.2% for *gfp* dsRNA‐injected SCB (Fig. 2). Statistical analyses showed that all mortalities were significant compared with *gfp* dsRNA‐injected SCB [*β‐actin* (*χ* ^*2*^ = 64.8, df = 1, *P* = 8E^−16^, *n* = 32), *α‐snap* (χ^2^ = 56.6, df = 1, *P* = 5E^−14^, *n* = 33), *rpt3* (*χ* ^*2*^ = 50.4, df = 1, *P* = 1E^−12^, *n* = 31), *rop* (*χ* ^*2*^ = 38.0, df = 1, *P* = 7E^−10^, *n* = 35), *α‐tubulin* (χ^2^ = 36.5, df = 1, *P* = 2E^−09^, *n* = 33), *srp54k* (*χ* ^*2*^ = 32.0, df = 1, *P* = 2E^−08^, *n* = 32) and *v‐ATPaseA* (*χ* ^*2*^ = 26.5, df = 1, *P* = 3E^−07^, *n* = 33)].

### 3.2 Feeding naked dsRNA to SCB results in weaker RNAi response and mortality

**Figure 3:**
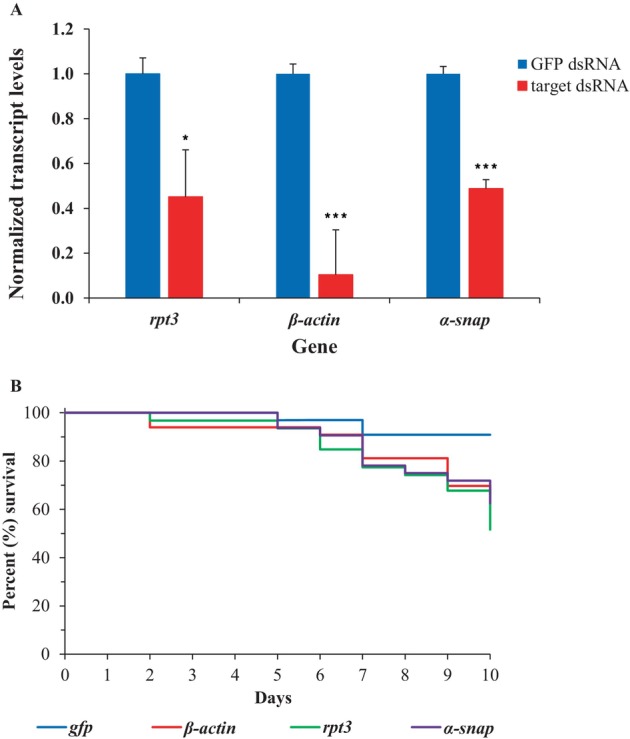
Gene knockdown and mortality in SCB fed with dsRNA. (A) Normalized transcript level was set to ‘1.0’ in *gfp* dsRNA‐fed SCB and relative transcript levels in target gene dsRNA‐fed SCB were calculated. Data are mean relative quantity ±SEM. Asterisks represent significant changes in the transcript levels in Student's *t*‐tests (*, *P* ≤ 0.05; ***, *P* ≤ 0.001), *n* = 3. (B) Kaplan–Meier survival curves showing the percent survival of SCB fed with dsRNA. Knockdown of *rpt3* (*n* = 31), *α‐snap* (*n* = 32) and *β‐actin* (*n* = 33) genes resulted in significant increases in mortality compared to control (*gfp, n* = 33) (*P* < 0.05).

Feeding naked dsRNA continuously to SCB adults resulted in significant gene knockdown for three genes compared with *gfp* dsRNA‐fed SCB [Fig. 3(A)]. Relative transcript levels were reduced by 54.8% (2.2‐fold), 51.2% (2.1‐fold) and 89.5% (9.6‐fold) for *rpt3, α‐snap* and *β‐actin*, respectively, all of which were significant [*rpt3 (t* = 5.1, *P* = 3.7E^−02^), *α‐snap* (*t* = 16.5, *P* = 4.8E^−04^) and *β‐actin (t* = 15.4, *P* = 1.1E^−04^)].

Feeding naked dsRNA also resulted in significant mortality in SCB compared with *gfp* dsRNA‐fed SCB. Gene knockdowns resulted in mortalities of 48.4% for *rpt3*, 37.5% for *α‐snap* and 33.3% for *β‐actin*, whereas mortality was 9.1% for *gfp* dsRNA‐fed insects [Fig. 3(B)]. Statistical analyses showed that all mortalities were significantly different than mortality in the *gfp* dsRNA‐fed control group [*rpt3* (χ^2^ = 11.5, df = 1, *P* = 7E^−04^, *n* = 31), *α‐snap* (χ^2^ = 7.0, df = 1, *P* = 8.00E^−03^, *n* = 32) and *β‐actin* (χ^2^ = 5.5, df = 1, *P* = 2.00E^−02^, *n* = 33)].

### 3.3 Feeding adult SCB dsRNA synthesized and delivered in *E. coli* results in weak RNAi response and no mortality

**Figure 4:**
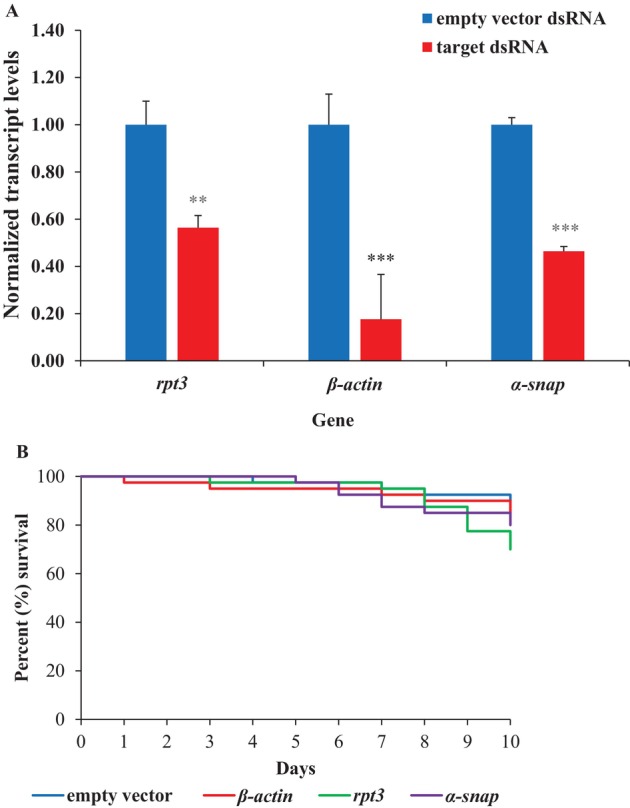
Gene knockdown and resulting mortality in SCB fed with *E. coli* encapsulated dsRNA. (A) Normalized transcript level was set to ‘1.0’ in empty vector dsRNA‐fed SCB and relative transcript levels in target gene dsRNA‐fed SCB were calculated. Data are mean relative quantity ±SEM. Asterisks represent significant changes in the transcript levels in Student's *t*‐tests (**, *P* ≤ 0.01; ***, *P* ≤ 0.001), *n* = 3. (B) Kaplan–Meier survival curves showing the percent survival of SCB fed with dsRNA. Knockdown of *rpt3* (*n* = 40), *α‐snap* (*n* = 40) and *β‐Actin* (*n* = 40) did not result in significant increases in mortality compared to control (*n* = 40), (*P* > 0.05).

As dsRNA synthesized and delivered in bacteria can improve oral RNAi in some insects,^42^, ^43^ dsRNA targeting *rpt3*, *α‐snap* and *β‐actin* also was produced in *E. coli* and fed to the insects. After 3 days of feeding, gene knockdown was 43.6% (1.8‐fold), 53.6% (2.2‐fold) and 82.4% (5.7‐fold) for *rpt3*, *α‐snap* and *β‐actin*, respectively [Fig. 4(A)]. Compared to empty vector dsRNA‐fed insects, gene knockdown was significant for all three genes [*rpt3* (*t* = 7.5, *P* = 1.7E^−03^), *α‐snap* (*t* = 27.8, *P* = 9.9E^−06^) and *β‐actin* (*t* = 11.1, *P* = 3.7E^−04^)]. However, no significant mortality was observed for any of the three genes targeted (*P* > 0.05) [Fig. 4(B)].

### 3.4 Tissue‐specific expression of dsRNase genes in adult SCB

**Figure 5:**
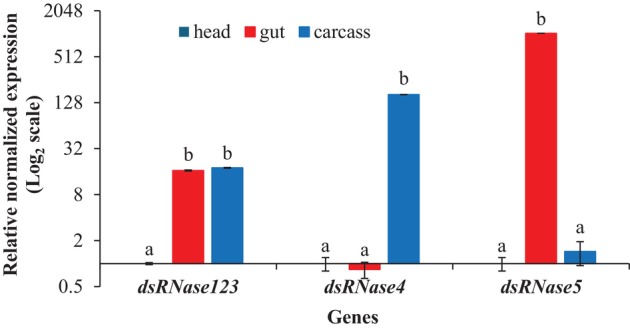
Tissue specific gene expression levels for dsRNase genes in SCB. Normalized expression was set to ‘1’ in the head and the relative fold‐change of each gene was calculated in other tissues. Bars represent mean relative quantity ±SEM, *n* = 3. Letters above bars denote significant differences between tissues. Means with the same letter are not significantly different (*P* > 0.05) according to Tukey's honestly significant difference tests (one‐way ANOVA).

*In silico* analysis of the SCB transcriptome revealed the presence of five distinct dsRNase sequences, referred to as *dsRNase1‐5* in this study (Table S3). As *dsRNase1‐3* sequences shared a highly conserved region with 100% identity, a single primer pair (referred to as *dsRNase123*) was designed to measure their combined expression simultaneously, whereas *dsRNase4* and *dsRNase5*, being more divergent, were analyzed separately. Results showed that combined expression of *dsRNase1‐3* was significantly higher in gut (16.6‐fold) and carcass (17.9‐fold) compared to head (*F*_2,6_ = 159.5, *P* = 6.3E^−06^), with no significant difference being found between gut and carcass (*P* ≥ 0.05). For *dsRNase4*, gene expression was significantly higher in carcass tissue compared to gut (193.8‐fold) and head (163.6‐fold) (*F*_2,6_ = 278.4, *P* = 1.2E^−06^), with no difference being observed between gut and head (*P* ≥ 0.05). As for the *dsRNase5* gene, expression was highest in gut tissue, which was significantly different from both head (~1038‐fold) and carcass (~718‐fold) (*F*_2,6_ = 246.9, *P* = 1.7E^−06^). There was no significant difference between head and carcass (*P* ≥ 0.05) (Fig. 5).

### 3.5 Exposure to dsRNA does not induce dsRNase genes

Expression of dsRNase genes was analyzed in insects injected with water or *gfp* dsRNA, and the results showed that none of the genes were induced in adult SCB (Fig. S1).

### 3.6 Effects of double knockdown of *dsRNase5* and *rpt3* on gene knockdown and mortality in adult SCB

**Figure 6:**
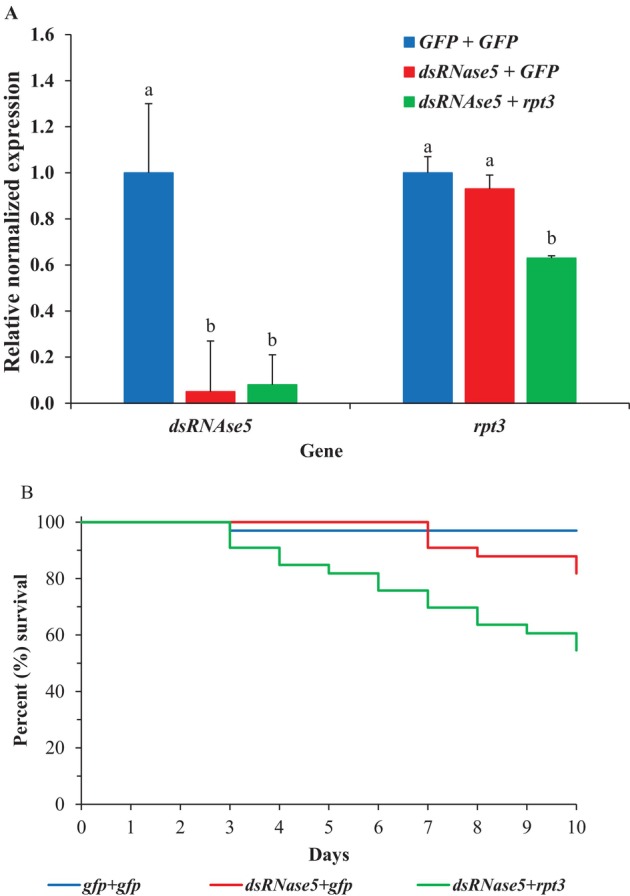
Normalized gene expression in SCB adults injected with dsRNase5 dsRNA and fed with rpt3 dsRNA to achieve double knockdown and resulting mortality. (A) The *gfp* + *gfp* treatment group served as the control, set to ‘1.0’. Transcript levels in the *dsRNase5* + *gfp* and *dsRNase5* + *rpt3* groups were calculated relative to the control. Data are mean relative quantity ±SEM, *n* = 3. Letters above bars denote significant differences between groups. Means with the same letter are not significantly different (*P* > 0.05) according to Tukey's honestly significant difference tests (one‐way ANOVA). (B) Kaplan–Meier survival curves showing the percentage survival of SCB in double knockdown experiments. Simultaneous knockdown of *dsRNase5* and *rpt3* genes resulted in a significant increase in mortality compared with the two control groups (*P* < 0.05, *n* = 33), with no difference observed between control groups.

*dsRNase5* was selected for double knockdown experiments to test if *dsRNase5* reduction would improve oral RNAi in SCB. Injection of *dsRNase5* dsRNA significantly reduced *dsRNase5* transcript levels by 94.8% (19.3‐fold) in *dsRNase5* + *gfp* and 91.3% (11.4‐fold) in *dsRNase5* + *rpt3* groups compared to *gfp* + *gfp* control (*F*_2,6_ = 90.6, *P* = 3.3E^−05^). Additionally, *rpt3* gene transcript levels were reduced significantly (36.5%, 1.6‐fold) only when *rpt3* dsRNA was fed to SCB compared to the two control groups (*F*_2,6_ = 21.2, *P* = 1.9E^−03^). No significant difference was observed between the two control groups (*P* ≥ 0.05) [Fig. 6(A)]. Furthermore, double knockdown of *dsRNase5* and *rpt3* resulted in significant mortality (45.5%) compared to the *gfp + gfp* and *dsRNase5 + gfp* control groups (χ^2^ = 19.1, df = 2, *P* = 7E^−05^, *n* = 33). No significant difference was observed between the two control groups (*P* > 0.05) [Fig. 6(B)].

### 3.7 dsRNA stability in SCB and CPB bodily fluids

**Figure 7:**
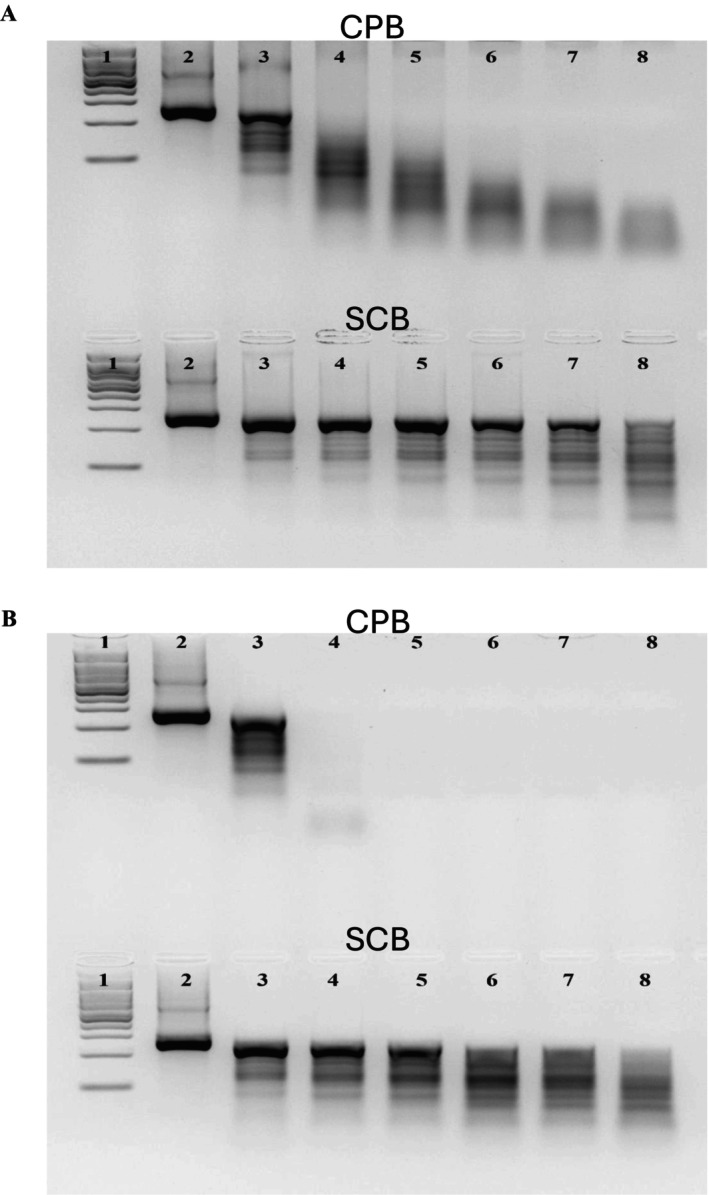
dsRNA degradation in SCB and CPB bodily fluids. (A) dsRNA degradation in SCB and CPB hemolymph. 1 = 100 bp DNA ladder; 2 = control (no hemolymph); 3 = 0 min; 4 = 30 min; 5 = 60 min; 6 = 120 min; 7 = 180 min and 8 = 300 min of dsRNA incubation with 10 μg protein. (B) dsRNA degradation in SCB and CPB digestive fluid. 1 = 100 bp DNA ladder; 2 = control (no digestive fluid); 3 = 0 min; 4 = 30 min; 5 = 60 min; 6 = 120 min; 7 = 180 min and 8 = 300 min of dsRNA incubation with 10 μg protein.

In order to explore mechanisms potentially limiting oral RNAi efficiency in SCB, dsRNA degradation in hemolymph and digestive fluid was compared between SCB and CPB, a coleopteran with extremely high oral RNAi efficiency. The results demonstrated that dsRNA was more stable in the bodily fluids of SCB compared to the bodily fluids of CPB. In CPB hemolymph, dsRNA started to be degraded immediately and only smearing of digested dsRNA was observed after 30 min of incubation. By contrast, with SCB hemolymph, the intact dsRNA was still visible at 300 min of incubation [Fig. 7(A)]. In CPB digestive fluid, dsRNA degradation was even greater, as dsRNA was completely degraded by 30 min, whereas in SCB digestive fluid, intact dsRNA was still visible at 180 min [Fig. 7(B)].

Adding 100 mm EDTA or heating digestive fluid at 100 °C for 10 min before incubations were started, completely eliminated dsRNA degradation (Fig. S2). Hemolymph pH in the two insects was similar—6.423 in CPB and 6.105 in SCB. For digestive fluid, however, there was a slight difference, with pH values of 4.704 in CPB and 5.407 in SCB.

### 3.8 Feeding naked dsRNA results in significant RNAi response in CPB

**Figure 8:**
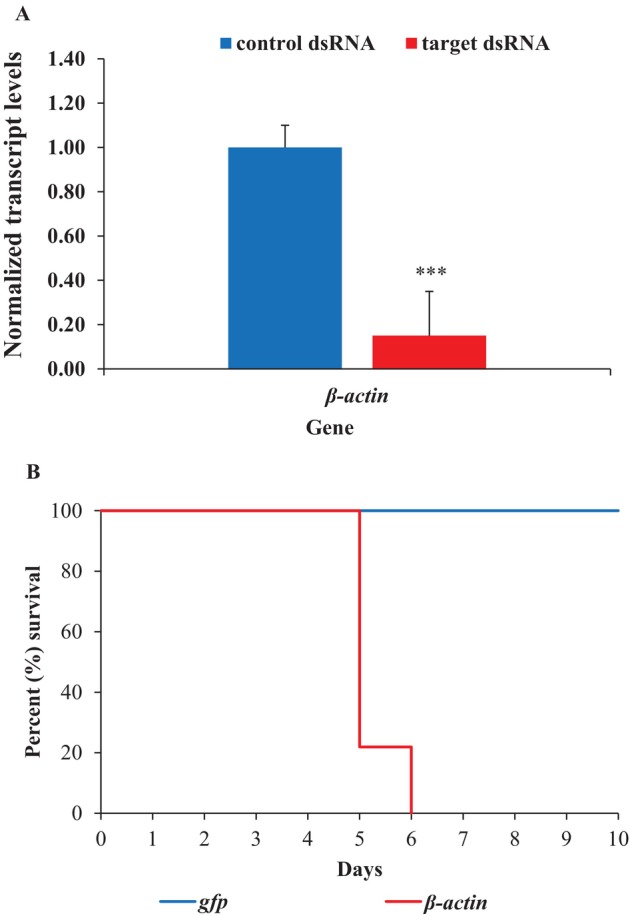
Gene knockdown and resulting mortality in CPB fed with naked dsRNA. (A) Normalized transcript level was set to ‘1.0’ in *gfp* dsRNA‐fed CPB and relative transcript levels in *β‐actin* dsRNA‐fed CPB were calculated. Data are mean relative quantity ±SEM. Asterisks represent significant changes in the transcript levels in Student's *t*‐tests (***, *P* ≤ 0.001), *n* = 3. (B) Kaplan–Meier survival curve showing the percent survival of CPB fed with dsRNA. Knockdown of the *β‐actin* gene (*n* = 3) resulted in a significant increase in mortality compared to control (*n* = 32) (*P* < 0.001).

In order to compare RNAi efficiency between SCB and CPB, the *β‐actin* gene was selected. For comparability, the CPB *β‐actin* dsRNA was designed to target the same region as in SCB. In CPB, feeding *β‐actin* dsRNA resulted in significant gene knockdown (84.7%, 6.5‐fold) compared to *gfp* dsRNA‐fed CPB (*t* = 8.7, *P* = 1.3E^−02^) [Fig. 8(A)]. Additionally, the gene knockdown resulted in 100% mortality in CPB [Fig. 8(B)] by the end of D6, which was highly significant (χ^2^ = 70.3, df = 1, *P* = 2.0E^−16^, *n* = 32). In fact, CPB fed with *β‐actin* dsRNA completely stopped feeding by the end of D3 and became moribund, followed by a sharp decline in survival, starting at D5.

### 3.9 *staufenC* expression in SCB and CPB

Expression of *staufenC*, a factor contributing to efficient RNAi in Coleoptera, did not differ significantly between the whole bodies of adult SCB and CPB (*P* ≥ 0.05) (Fig. S3). Analysis of *staufenC* mRNA levels in different tissues in SCB showed gut tissue had significantly higher expression compared to head and carcass (*F*_2,6_ = 39.5, *P* = 3.5E^−04^) (Fig. S4). No significant difference was observed between head and carcass (*P* ≥ 0.05).

## 4 DISCUSSION

RNAi is considered a promising technology for pest management owing to its specificity. However, its ineffectiveness in some insects hinders its use as an alternative to conventional insecticides. Even in coleopteran insects, which are considered the most susceptible, RNAi efficacy varies,^32^ underscoring the importance of assessing each species for RNAi susceptibility. This study focused on analyzing RNAi susceptibility of the coleopteran pest species, SCB.

First, we verified that SCB possesses the molecular machinery required to trigger a robust RNAi response via injection of dsRNA targeting seven genes, resulting in significant mRNA transcript reductions for all genes targeted. The most efficient gene target was *β‐actin*, whose knockdown resulted in 100% mortality by D9 postinjection. Multiple studies have shown *β‐actin* as a highly efficient RNAi target.^16^, ^19^, ^44^, ^45^ The next most efficient targets were *α‐snap* and *rpt3*, both of which led to mortalities ≥90%. We observed that percentage gene knockdown efficiency did not always correlate well with percentage mortality. For instance, for the *v‐ATPaseA* gene, whereas gene knockdown was the second highest, mortality was the lowest. By contrast, for the *rpt3* gene, gene knockdown was lowest, whereas mortality was third highest. A similar lack of correlation between the efficiency of gene knockdown and lethal phenotype also has been observed in other insects.^46^, ^47^ Furthermore, some gene targets producing 100% RNAi mortality in other coleopteran species, produced only moderate mortality in SCB. For example, the *srp54k* and *rop* genes resulted in 100% mortality in *P. cochleariae* ^24^ and *T. castaneum*,^48^ but only 71% and 77% mortality in SCB, respectively. This illustrates how the efficiency of RNAi targets varies among insects and why multiple genes should be tested to identify the best targets in each species.

In order to evaluate oral RNAi efficiency, we selected the three most effective gene targets (*β‐actin*, *α‐snap* and *rpt3*) from injections and fed naked dsRNA to SCB. For all three targets, both gene knockdown and resulting mortalities were significantly higher than for insects fed with *gfp*‐dsRNA, but were considerably lower than with injections. For instance, whereas injections resulted in 99.4% transcript reduction and 100% mortality for *β‐actin*, oral delivery of the same naked dsRNA resulted only in 89.5% gene knockdown and 33.3% mortality. This is noteworthy, as it implies that very strong knockdown is required for a lethal phenotype in SCB using *β‐actin*. By contrast, gene knockdown of 51.2% and 54.8% for *α‐snap* and *rpt3* resulted in 37.5% and 48.4% mortality, respectively. Despite having the lowest gene knockdown efficiency among the three targets, *rpt3* was the best target for orally delivered naked dsRNA, as it resulted in highest mortality. Weaker phenotypic effects from oral delivery of naked dsRNA, as opposed to injection, has also been observed with other coleopterans^49^, ^50^ and other insect orders.^51^, ^52^

Delivering dsRNA in bacteria or nanomaterials can improve RNAi efficiency,^53^, ^54^, ^55^ presumably as a result of protection from nucleases afforded by the cellular or nanomaterial carrier. For instance, in CPB, synthesizing and delivering dsRNA in bacteria previously has been shown to yield more efficient gene knockdown for several genes compared to naked dsRNA.^25^ Therefore, we fed SCB with *β‐actin*, *α‐snap* and *rpt3* dsRNA contained in bacteria, which resulted in significant gene knockdowns, but not significant mortality. This lack of mortality might be a consequence of the fact that the level of gene knockdown using dsRNA delivered in bacteria, although significant, was not as efficient as it was in the naked dsRNA‐fed group. For example, with *rpt3*, which had the highest level of mortality with naked dsRNA (48.4%), the drop in knockdown efficiency resulting from bacterial delivery was the largest, falling from 54.8% to only 43.6% silencing, a difference of 11.2%. Even small changes in knockdown efficiency can have large effects on the rate of mortality and this reduction in levels of silencing with bacterially produced dsRNA may have been adequate to lower mortality below the level of significance. Because protection of dsRNA within bacterial cells failed to improve silencing efficiency, this suggests that dsRNA degradation is not the reason for lower silencing by dsRNA feeding *versus* injection.

dsRNases in insect guts have been shown to diminish RNAi efficiency in numerous insects, including Coleoptera.^56^, ^57^, ^58^, ^59^ In SCB, we identified five distinct dsRNase genes, of which, d*sRNase5* exhibited the highest expression in the gut. However, our results showed that *rpt3* knockdown was less efficient when combined with *dsRNase5* knockdown than it was in the naked dsRNA‐fed group. The failure of dsRNase reduction to increase silencing efficiency again suggests that dsRNA degradation is not limiting silencing by dsRNA feeding in SCB. Lack of improvement in RNAi response following dsRNase knockdown also has been observed in the desert locust, *Schistocerca gregaria* (Forskål), in which double knockdown of four *dsRNases* did not enhance RNAi efficiency of a marker gene.^58^ Additionally, in the fruit fly, *Drosophila melanogaster* (Meigen), knocking out the dsRNA degrading *snipper* gene does not improve RNAi.^60^ In our study, we could not simultaneously knockdown both *dsRNase5* and *rpt3* using oral delivery, because silencing by feeding is not efficient enough to strongly silence the chosen dsRNase. We specifically targeted *dsRNase5* in the double knockdown because it was the gene most highly expressed in the gut. However, it also is possible that a different dsRNase, one induced by dsRNA exposure, might be important in degradation of ingested dsRNA. Therefore, we also tested all three dsRNase genes to check if any of them is induced by dsRNA exposure, but found no significant induction of any of the dsRNase genes (Fig. S1).

The coleopteran exhibiting perhaps the greatest sensitivity to RNAi and which is often used as a model for its study, is the CPB. Therefore, physiological properties relevant to RNAi in SCB were compared with those of CPB, to reveal possible causes for the low level of oral RNAi efficiency in SCB. As dsRNases exhibit higher activity in alkaline environments,^61^ we confirmed that the pH of bodily fluids from SCB and CPB were acidic. The hemolymph from both insects had comparable pH values, consistent with previously reported CPB hemolymph values.^62^ In the digestive fluid, however, the pH was slightly higher in SCB. Nevertheless, the overall pH in both bodily fluids was acidic in both insects and therefore would not be expected to significantly influence dsRNase activity.

For dsRNA degrading activities in bodily fluids, our results demonstrated that dsRNA was more stable in both hemolymph and digestive fluid of SCB compared to those of CPB. Additionally, for both insects, adding EDTA or heating inhibited dsRNA degradation in the digestive fluid. Although surprising, the greater nuclease activity of CPB hemolymph and digestive fluid aligns with a previous study, which showed that CPB bodily fluid degrades dsRNA at lower concentrations than SCB bodily fluid.^63^ Interestingly, the same study also demonstrated that CPB, compared to SCB, converts orally‐delivered dsRNA into siRNA molecules more efficiently, and is likely to contribute to its greater RNAi sensitivity.

In coleopteran insects, conversion of dsRNA into siRNA requires StaufenC,^31^, ^64^, ^65^ and our results showed that transcript levels of the *staufenC* gene do not differ between the two insects. Furthermore, expression of *staufenC* is significantly higher in the gut tissue in SCB compared to head and carcass, which is a similar distribution to that found previously for CPB^66^. However, comparing orally delivered RNAi efficiency between SCB and CPB showed a large difference when each was fed dsRNA targeting *β‐actin*. In CPB, feeding of *β‐actin* dsRNA led to a complete cessation of feeding by the end of D3, followed by a sharp decline in survival and subsequent 100% mortality, similar to what was observed by Zhang *et al*. (2025) when *β‐actin* dsRNA was delivered orally in transplastomic potato.^16^ By contrast, mortality was limited to just 33.3% in SCB. Taken together, our results suggest that factors other than StaufenC or the activities of dsRNases in the gut, might be limiting oral RNAi response in SCB.

One possible explanation for the modest oral RNAi response in SCB may be the influence of its gut microbiome, as recent studies have highlighted the role of gut microbiome in RNAi response in other insects.^66^, ^67^ As part of their life cycle, SCB larvae feed on plant roots, whereas adults feed on leaves, flowers and pollen.^1^ This complex life cycle undoubtedly exposes SCB to diverse microorganisms such as bacteria, fungi and viruses, which can colonize the insects and establish symbiotic or pathogenic relationships with the host. In fact, a key difference between SCB and CPB is that SCB is known to vector plant pathogens,^68^ whereas CPB mainly causes plant damage through leaf defoliation.^69^ This distinction may help explain the differences in oral RNAi response between these two coleopteran insects. As we have demonstrated, neither differences in *staufenC* expression nor dsRNA degradation by dsRNases appear to be the reason for inefficient oral RNAi in SCB. Therefore, other factors, such as the gut microbiome or other endosymbiotic organisms, may play a role in RNAi response in SCB. The presence of complex barriers to efficient RNAi in SCB will affect the future potential of RNAi‐based strategies for control of this pest. Further investigation of the basis of these barriers will be needed before application of RNAi can be realized with SCB.

## 5 CONCLUSIONS

Our results using dsRNA injections demonstrate that SCB possesses the machinery to mount a robust RNAi response; however, the response is limited for oral RNAi. Neither dsRNA degradation in the gut nor a lack of *staufenC* expression appear to be limiting factors in oral RNAi response in this species, suggesting that other factors influence oral RNAi response in SCB.

## FUNDING INFORMATION

This project was funded by Agriculture & Agri‐Food Canada project no. 4956.

## CONFLICT OF INTEREST

The authors declare no competing interests.

## ETHICS STATEMENT

No approval was required as experiments were conducted with an unregulated invertebrate species.

## AUTHOR CONTRIBUTIONS

EK and CD conceived and designed the experiments; EK conducted the experiments and analyzed the data; EK and CD wrote the manuscript; FL reared SCB and provided technical assistance for injections; all authors read and approved the final manuscript.

## Supporting information


**Table S1. List of primers used in dsRNA synthesis**.
**Table S2. List of qRT‐PCR primers used for gene expression analysis**.
**Table S3. Gene sequences used in this study**.
**Fig. S1. *dsRNase* gene expression in insects injected with water or *gfp* dsRNA**. Normalized transcript level was set to ‘1.0’ in water‐injected SCB and relative transcript levels in *gfp* dsRNA‐injected SCB were calculated. Data are mean relative quantity ±SEM, *n* = 3. No significant changes in the transcript levels were found in Student's *t*‐tests (*P* ≥ 0.05).
**Fig. S2. Elimination of dsRNA degradation in SCB and CPB digestive fluids. (A)** Samples were heated to 100 °C before dsRNA incubation. **(B)** 100 mm EDTA was added to the reaction mixtures. 1 = 100 bp DNA ladder; 2 = 0% (control) digestive fluid; 3 = 5.00%; 4 = 2.50%; 5 = 1.25%; 6 = 0.625%; 7 = 0.312%; 8 = 0.156%; 9 = 0.078% and 10 = 0.039% of digestive fluid.
**Fig. S3. *staufenC* gene transcript levels in whole bodies of SCB and CPB**. Normalized transcript levels were set to ‘1.0’ in CPB and relative transcript levels in SCB were calculated. Data are mean relative quantity ± SEM. No significant differences between the two insects were found in Student's *t*‐tests (*P* ≥ 0.05), *n* = 3.
**Fig. S4. *staufenC* gene transcript levels in different tissues of SCB**. Normalized expression was set to ‘1’ in the gut and the relative fold‐change of each gene was calculated in other tissues. Bars represent mean relative quantity ±SEM, *n* = 3. Letters above bars denote significant differences between tissues. Means with the same letter are not significantly different (*P* > 0.05) according to Tukey's HSD tests (one‐way ANOVA).

## Data Availability

The data that support the findings of this study are available from the corresponding author upon reasonable request.
