# Supplementary material for: Evaluating the potential of RNA interference for control of striped cucumber beetle, Acalymma vittatum (Fabricius) (Coleoptera: Chrysomelidae)
Source: Pest Manag Sci. 2025 Dec 25;82(4):3591–602. doi: 10.1002/ps.70481 (PMC12976179; doi:10.1002/ps.70481)
Supplement: Supplementary file 1 — Table S1. List of primers used in dsRNA synthesis. Table S2. List of qRT‐PCR primers used for gene expression analysis. Table S3. Gene sequences used in this study. Fig. S1. dsRNase gene expression in insects injected with water or gfp dsRNA. Normalized transcript level was set to ‘1.0’ in water‐injected SCB and relative transcript levels in gfp dsRNA‐injected SCB were calculated. Data are mean relative quantity ±SEM, n = 3. No significant changes in the transcript levels were found in Student's t‐tests (P ≥ 0.05). Fig. S2. Elimination of dsRNA degradation in SCB and CPB digestive fluids. (A) Samples were heated to 100 °C before dsRNA incubation. (B) 100 mm EDTA was added to the reaction mixtures. 1 = 100 bp DNA ladder; 2 = 0% (control) digestive fluid; 3 = 5.00%; 4 = 2.50%; 5 = 1.25%; 6 = 0.625%; 7 = 0.312%; 8 = 0.156%; 9 = 0.078% and 10 = 0.039% of digestive fluid. Fig. S3. staufenC gene transcript levels in whole bodies of SCB and CPB. Normalized transcript levels were set to ‘1.0’ in CPB and relative transcript levels in SCB were calculated. Data are mean relative quantity ± SEM. No significant differences between the two insects were found in Student's t‐tests (P ≥ 0.05), n = 3. Fig. S4. staufenC gene transcript levels in different tissues of SCB. Normalized expression was set to ‘1’ in the gut and the relative fold‐change of each gene was calculated in other tissues. Bars represent mean relative quantity ±SEM, n = 3. Letters above bars denote significant differences between tissues. Means with the same letter are not significantly different (P > 0.05) according to Tukey's HSD tests (one‐way ANOVA). [file PS-82-3591-s001.docx]

**Supplementary file for SCB paper**

**Table S1. List of primers used in dsRNA synthesis.**

| **Gene** | **Forward and Reverse primers**  **(5’ -3’)^*^** | **Amplicon size (bp)** |
| --- | --- | --- |
| *β-actin* | ATGACGCACCCCGTGCCGT  CGTACAAGGAGAGTACGGCTT | 360 |
| *α-tubulin* | TTCTTGGGGAGCTGGGTAGA  TGTCCCGAGAGCAGTATTCG | 349 |
| *v-ATPaseA* | TTCTATGAACGTGCAGGTCG  CTTCTTTAAGGAGCCTGGCA | 400 |
| *rop* | GCATCTGCAGGAGTGTTGAA  TTGTCCTTGATTTGAGCGTG | 496 |
| *rpt3* | CATGCTCCAAGCTGATGAAA  CACTTCACGATCAGCACCTG | 400 |
| *α-snap* | ACACTGAAATGGGTCGCTTT  GAATCTTGAAATGCGGGGTA | 382 |
| *srp54k* | TCTCGCGGATTTAGGTCGTA  ACTTGGTCATAGGCACCAGC | 438 |
| *dsRNase5* | ATAGCGAATGTTTTGACGCC  GTCCATAAGTTCCCGTCCAA | 513 |
| *gfp* | ACTTTTCACTGGAGTTGTCCCAA  AGTAGTGACAAGTGTTGGCTGA | 223 |
| CPB *β-actin* | CACGAGGTTTTTCTGTCTAG  ATGTCATCCCAGTTGGTGAT | 296 |

**^*^**T7 promoter sequence was added to 5’ends of the primers for *in vitro* dsRNA synthesis.

**Table S2. List of RT-qPCR primers used for gene expression analysis.**

|  | **SCB RT-qPCR primers** |  |  |
| --- | --- | --- | --- |
| **Gene** | **Forward and Reverse primers (5’ -3’)** | **Amplicon size (bp)** | **Efficiency (%)** |
| *ef1α* | CAATTTCAGGATGGCACGG  AGCAGGAGGTAAGATGGCA | 143 | 91.2 |
| *rpl8* | AACTGCTGGCCTAAGGTAC  TTACGACCAGCAGAGGTTC | 122 | 91.2 |
| *β-actin* | ACTTGGCTGGTCGTGACTT  GAGGGCAACATAGCAGAGT | 128 | 90.4 |
| *α-tubulin* | TTTGCCAGCACCTGTTTCACT  TGCTAAAGCCTGCTGGGAAT | 133 | 90.0 |
| *v-ATPaseA* | TGATGGCTGACTCCACTTCAC  CAAACGGGCACCCAAGTAAG | 110 | 91.4 |
| *Rop* | GCTGCTAGGAATGCCAATATGG  GAGCCAACTCAACGTTTCTTTCC | 121 | 95.6 |
| *rpt3* | TCGGAAGTAAAGCCAGTGCT  CTCCTGGGCATGTAAATACTCT | 156 | 90.7 |
| *α-snap* | GCATAGAGGATGCAGTGGAATG  GACGGGCTCCAGTTCTTGC | 126 | 94.4 |
| *srp54k* | CGGCAGAGTGGTGAGAGTTG  GCCGCCTTTGAAGAGACCTT | 133 | 96.6 |
| *dsRNase123* | GGAACAGGTAAGGCTTTGGTT  TGGACTGGTACTTATGGGATAG | 104 | 96.6 |
| *dsRNase4* | CTGTTGGAACCATAATGTAGGC  ACGAAAGCGTCTATGGGCT | 100 | 93.2 |
| *dsRNase5* | TTTCGAGCCTTCCTCACTCT  TTTAGGTACTGGCAACGCAG | 101 | 97.4 |
| *staufenC* | TAGCTGCACAGGCCCTAGAT | 91 | 92.4 |
|  | TTCTTGCATTCGGCCTCAGA |  |  |
|  | **CPB RT-qPCR primers** |  |  |
| **Gene** | **Forward and Reverse primers (5’ -3’)** | **Amplicon size (bp)** | **Efficiency (%)** |
| *ef1α* | CAGGGCAAGGTTTGAAAGATAA  CCATCAGCACAGTTCCCAT | 168 | 99.6 |
| *l8e* | GGTAACCATCAACACATTGG  TCTTGGCATCCACTTTACC | 124 | 97.4 |
| *β-actin* | GCAGAAGGAAATCACCGCTC  GGACAGGGAAGCCAAGATG | 112 | 91.7 |
| **staufenC* | GGGTGTTTCATACCTCGTCTC | 107 | 92.5 |
|  | CCCTTACACTGGGAACAGAAA |  |  |

*****CPB StauC primers were taken from Yoon et al. 2018 (Reference 31 of manuscript).

**Table S3. Gene sequences used in this study.**

|  | **SCB gene sequences** |
| --- | --- |
| **Gene** | **Sequence** |
| SCB_*rpl8* (reference gene) | AACTACGTTATTTAGATTTTTCAGAATGACATGGTTACATTAAGGGTGTTGTTAAAGTTATCATACATGACCCTGGCCGTGGTGCTCCATTGGCAGTAGTACATTTCAGAGATCCCTACAAGTTCCAAACAAGGAAAGAATTATTTATTGCCCCCTAAGGAATGTACACTGGACAATTCTTGTACTGTGGCAAAAAAGCCAACCTCCAAATTGGAAACGTATTGCCTGTAGGAACTATGCCTGAAGGTACAATTGTATGCAATTAAGAAGAGAAAACTGGTGACCGTGGTCGGTTAGCACGTGCATCTGGAAATTATGCTACAGTAATTGCCCACAATCACGACACAAAGAAAACCAGAGTTAAGTTGCCTTCTGGAGCCAAAAAAGTTATTCCATCTAACAACAGGGCTATGGTTTGTATCGTTGCTGGAGGTGGTCGTATTGACAAACCAATCTTGAAAGCAGGTCGTGCTTACCATAAATACAAGGTTAAACGTAACTGCTGGCCTAAGGTACGTGGTGTTGCTATTAACCCCGTTGAACATCCTCACGGAGGTTGTAACCATCAACACATTGGTAAAGCATCTACAGTTAAGAGAGGAACCTCTGCTGGTCGTAAAGTTTGTCTTATTGCTGCCAGGAGAACTGGTAGGATTAGGGG |
| SCB_*ef1α* (reference gene) | AACCAGCATACAGCGAATCACGTTTTGAGGAAATCAAGAAGGAAGTATCCTCATACATCAAAAAGATTGGTTACAACCCAGCCGCTGTTGCCTTCGTACCAATTTCAGGATGGCACGGTGATAACATGTTAGAAGTATCTGAGAAGATGCCATGGTTCAAGGGATGGGCCATTGAACGTAAAGAAGGAAAGGCTGAAGGAAAATGTTTTATTGATGCTTTAGATGCCATCTTACCTCCTGCTCGTCCAACTGACAAACCCCTCCGTCTTCCACTCCAGGATGTCTACAAAATTGGTGGTATTGGAACAGTACCTGTTGGTCGTGTTGAAACTGGTGTTTTGAAACCTGGTATGGTTGTAGTATTTGCCCCAGCCAATTTGACCACTGAAGTAAAATCCGTTGAAATGCACCACGAAGCCCTCCCAGAAGCTGTACCCGGAGACAATGTAGGTTTCAACGTTAAGAACGTTTCTGTTAAAGAATTGCGTCGTGGTTACGTAGCTGGAGACTCCAAGAACAACCCACCCAGGGGAGCTTCAGACTTCCTTGCCCAAGTCATTGTACTCAATCACCCCGGTCAAATTTCCAATGGTTACACTCCCGTGCTCGACTGTCACACAGCCCACATCGCCTGTAAATTCGCTGAAATCAAAGAAAAGGTTGACCGTCGTTCTGGTAAAACTACTGAAGAAAATCCCAAAGCCATTAAAACTGGAGATGCCGCCATTGTCAACTTGGTACCCACCAAGCCAATGTGTGTAGAATCATTCCAAGAATTCCCACCACTTGGACGTTTTGCAGTCCGTGACATGAGGCAGACTGTTGCTGTAGGAGTTATCAAGAGTGTTGCCTTCAAAGATCCTTCCTCCGGAAAAGTCACAAAAGCCGCAGAAAAGGCCCAAAAGAAGAAATAGTCTGCTCGAACTCCATTACTTAATAATTTTGATATGTTTTGTTTCTTTTATATAATAATATTGGAGTATCAATGTATTTTTAATAACTAAAGGACTTTTGCATTGCATTCTTTGTGATCTGTAAATTAAACCTGGACAAATAAAGAC |
| SCB_*β-actin* | ATGACGCACCCCGTGCCGTCTTCCGCTCAATCGTCGGTCGCCCAAGGCATCAAGGTGTAATGGTTGGTATGGGACAAAAAGACTCATACGTTGGAGATGAAGCCCAAAGCAAAAGAGGTATCCTCACCTTGAAATACCCAATTGAACACGGTATCATCACCAACTGGGATGATATGGAAAAGATCTGGCATCACACCTTCTACAATGAACTCCGTGTTGCTCCAGAAGAACACCCAGTCCTCCTCACTGAAGCTCCACTCAACCCCAAGGCCAACAGAGAAAAGATGACCCAAATCATGTTTGAAACCTTCAACACACCCGCCATGTATGTAGCCATCCAAGCCGTACTCTCCTTGTACGCTTCCGGTCGTACCACAGGTATTGTATTGGACTCTGGAGATGGTGTCACCCACACCGTACCAATCTACGAAGGTTACGCTCTTCCCCACGCCATCCTCCGTTTGGACTTGGCTGGTCGTGACTTGACCGACTACCTCATGAAAATCCTTACCGAAAGAGGTTACTCATTCACCACCACCGCTGAAAGGGAAATTGTTCGTGACATCAAGGAAAAACTCTGCTATGTTGCCCTCGACTTC |
| SCB_*α-Tubulin* | GTGGGGGGTCAAGATAGAGTTGTATGGTTCTACTACAGCAGTAGATTCTTGGGGAGCTGGGTAGATGGCTAATTCAAGCTTGGATTTTTTACCATAGTCAACAGAGAGACGTTCCATCAACATAGAAGTGAAGCCGGATCCAGTACCACCACCAAATGAGTGGAAGATCAAAAAGACTTGTAGTCCAGTACATTGATCGGCCAGTTTACGGATTCTGTCCAATACCAAGTCAACTATTTCTTTACCAATTGTATAGTGACCTCTGGCGTAATTGTTGGCAGCGTCTTCTTTGCCTGTGATGAGTTGTTCAGGGTGGAACAACTGACGGTAGGTACCAGTACGTACTTCATCTACTACTGTTGGTTCAAGATCTACGAATACTGCTCTCGGGACATGTTTGCCAGCACCTGTTTCACTGAAGAAGGTGTTGTAAATGTCATCTCCTCCTCCAACAGTTTTGTCAGCAGGCATCTGACCATCAGGTTGAATTCCATGTTCCAGTCAGTATAATTCCCAGCAGGCTTTAGCAATGTGGACTACGGCTTGGCCGACATTGACTGAGATAAATTCAAGCATTTTGTTTAATGAGTT |
| SCB_*v-ATPAseA* | ACTGAATCTATTATGAAGCGTACCGCATTGGTCGCCAATACCTCCAACATGCCTGTAGCTGCTCGTGAAGCTTCTATCTATACTGGTATTACCCTTTCTGAGTACTTCCGTGATATGGGTTACAATGTATCTATGATGGCTGACTCCACTTCACGTTGGGCCGAAGCTTTGAGAGAAATTTCAGGTCGTTTGGCTGAAATGCCTGCCGATTCCGGTTACCCGGCTTACTTGGGTGCCCGTTTGGCTTCCTTCTATGAACGTGCAGGTCGTGTTAAATGTCTAGGTAACCCAGATAGAGAAGGATCCGTTTCAATTGTAGGAGCTGTATCTCCTCCTGGTGGTGACTTCTCAGATCCTGTTACCACCGCCACTCTTGGTATTGTACAGGTGTTCTGGGGTTTGGACAAGAAATTAGCCCAACGTAAGCACTTCCCTTCTGTAGATTGGCTTGGTTCCTACTCCAAATATTTAAGAGCATTGGATGACTTCTATGACAAAAACTTCCAAGAGTTTATACCTCTTAGAACCAAAGTTAAGGAAATTCTTCAGGAAGAAGATGATCTAGCCGAAATTGTGCAGCTGGTAGGTAAAGCATCTCTAGCAGAAACAGACAAAATTACTTTGGAAATTGCCAGGCTCCTTAAAGAAGATTTCTTGCAACAAAACTCGTACTCTTCCTACGATAGATTCTGTCCATTTtACAAAACCGTTGGTATGCTTAGAAACATGATTGGTTTGTATGACATGGCAAGACATGCTGTAGAATCAACCGCAC |
| SCB_*rop* | ATGAATGAAGTCATCAAGCACAAGCCTACCAAGAAGAACGGGCCAACCCCTGGACAGCAAGCTCATGGAGTAGAGTGGAGAATTCTCGTAGTAGACCAGCTTGCAATGAGGATGGTTTCAGCGTGTTGCAAGATGCATGATATATCGGCAGAAGGAATAACATTGGTTGAGGATATTATGAAGAAAAGGGAACCCCTTGGAACTATGGAAGCTGTGTACCTGATAACTCCTTCAGAAAAGTCCGTTCATGCATTGATGAATGACTTTGAACCTCCAAGACAGATGTACAGAGGTGCACATGTATTTTTCACAGAAGCGTGTCCAGACGAATTATTTAACACACTGTGCCACCATCCAGTAGCAAAATTTATTAAAACTCTAAAAGAAATCAACATAGCATTCATTCCGACTGAGTCACAGGTATTGAAAATTGCTTAAAAACACTTACAGGTATTCTCGCTGGACTCACCAGACACGTTCCAATGTAGTTATGATCCATCATTTTCCGCTGCTAGGAATGCCAATATGGAAAGGATGGCTGAACAAATTGCAACTCTTTGCGCAACACTCGGGGAATACCCGCATGTCAGATATAGAAGTGATTGGGAAAGAAACGTTGAGTTGGCTCAACTAATTCAGCAAAAGCTGGACGCATACAAAGCCGATGAACCTACCATGGGAGAGGGACCAGAAAAGGCAAGATCACAGTTACTCATCCTTGATCGAGGTTTCGACTGTGTGTCGCCTCTCCTCCACGAACTCACCTTCCAGGCAATGGCCTATGACTTATTACCCATAGAAAACGACGTCTATAAGTACGAAGCATCTGCAGGAGTGTTGAAGGAAGTCCTCTTGGACGAAAACGACGAACTGTGGGTGGATTTACGTCATCAACACATCGCTGTAGTGTCTCAAAGTGTTACGAAGAATCTGAAGAAATTCACAGACTCCAAACGCATGACTCAGAGTGACAAGCAATCGATGAAAGATCTCTCCACCATGATCAAGAAGATGCCGCAGTACCAGAAGGAATTGTCCAAGTATGCTACCCATCTTCACCTGGCTGAAGACTGCATGAAGGCCTATCAGGGATATATCGACAAGTTATGCAAAGTTGAACAGGACTTGGCCATGGGAACGGACGCAGAAGGCGAGAAAATCAAGGATCACATGCGCAACATCGTGCCTATCTTACTGGATCCCAAAATTAGCAATGAGTACGACAAAATGCGTATTATAGCGTTGTAGCAATGACGAAAAACGGCATTACAGACGAAAATCTGTCAAAATTGGCTACTCACGCTCAAATCAAGGACAAACTTACCATCGCCAACCTTCAGTTACTTGGAGTCAACGTTATTAACGACGGCGGCCCAAGGAAAAAGCAGTATACAGTGCCGCGCAAAGAGAGGATTACGGAACAGACGTACCAAATGTCCAGATGGACGCCCGTCATTAAGGATATAATGGAGGATTGCATAGACGACAAACTGGATCAGAAACACTTCCCGTATTTGAGTGGGAGAGCACAATCCACTGGATACCATGCAGCGCCTTCCAGTGCCCGTTACGGCCAGTGGCACAAAGACAGAGGCCAGCAAGCCGTCAAAAACGTCCCCCGTCTGATCGTCTTCGTCGTGGGAGGGATCAGCTTCTCAGAGATACGATGCGCGTACGAAGTGACCAACGCCCAGAAAAACTGGGAAGTCATCATCGGCTCGTCGCACATACTCACCCCCGAAGACTTCCTAAGCAATCTGGCAACGTTGGCCGGCTAG |
| SCB_*rpt3* | ATGGAAGCTATGGATATAGTTATACCTGAAAAAGAAGAAGGTAGCCCTTCGGAAGTAAAGCCAGTGCTACAGGATCTGGATACTGAAGATTTATATACGAAGTATAAAAAACTTCAACAGCAGTTAGAATTCTTGGAGGTTCAAGAAGAGTACATTAAAGATGAGCAAAGGAATCTGAAGAAAGAGTATTTACATGCCCAGGAGGAAGTTAAACGTATTCAGTCCGTCCCCTTAGTTATTGGACAATTTTTAGAAGCAGTAGATCAGAACACAGGCATTGTGGGCTCAACTACAGGTTCAAATTATTATGTTAGAATATTATCAACAATCGACAGAGAACTTTTAAAACCTTCTGCTAGTGTTGCCCTTCATAAGCACAGCAATGCCTTAGTAGATGTCCTACCTCCAGAGGCAGATTCTTCAATAAGCATGCTCCAAGCTGATGAAAAACCTGATGTTCAATACAGCGATATTGGAGGCATGGATATGCAGAAACAAGAAATTAGAGAAGCTGTGGAATTACCTTTGACTCACTTTGAATTGTACAAACAGATTGGTATTGATCCTCCCAGAGGTGTCCTGATGTATGGACCTCCTGGATGTGGCAAAACAATGTTGGCTAAGGCTGTTGCTCATCATACTACAGCCGCATTTATTCGTGTGGTTGGATCAGAATTCGTACAGAAATATCTTGGTGAAGGTCCCAGAATGGTCAGAGATGTGTTCCGTTTGGCAAAAGAAAATTCTCCGGCAATCATTTTTATTGATGAAATTGATGCAATTGCAACTAAACGTTTTGATGCTCAGACAGGTGCTGATCGTGAAGTGCAGAGAATTTTGTTGGAATTACTTAACCAGATGGATGGTTTCGATCAAACAACAAATGTTAAGGTAATAATGGCAACTAACAGGGCTGATACCTTAGATCCTGCACTTTTGCGACCTGGTCGTCTCGACAGAAAAATTGAATTTCCTTTACCAGATAGAAGACAGAAGCGTTTGATTTTCAGCACTATCACATCTAAAATGAACTTATCAGAGGAGGTCGACTTGGAGGACTATGTTGCAAGACCAGATAGAATATCAGGAGCGGACATCAACGCCATCTGCCAAGAAGCCGGTATGCATGCTGTTCGAGAAAACCGTTACATCGTTTTGCCTAAAGATTTTGAGAAGGGTTATAAGAATAATATTAAGAAAGATGAGAGTGAACACGAGTTTTACAAATAA |
| SCB_*α-snap* | ATGCATTCAAAGATTCCAATAGCTCAGCAGTTAATGGCACAGGCAGAATATATAGCATGTTCCGTTGAAGCTTTCTTTATATATTTATTTCGTGGATCAAGCCGCATAGAGGATGCAGTGGAATGTTACACAAGAGCTGCAAATCTTTTCAAAATGGCCAAAAGTTGGGACGCTGCAGGTAAAGCATTTTGTGAGGCTGCAAATTTACATGCAAGAACTGGAGCCCGTCATGATGCTGCCACCAATTACATAGATGCTGCAAATTGTTACAAAAAAGCTGATGTATTTGAGGCTGTAAATTGCTTCATAAAAGCTATAGATATCTACACTGAAATGGGTCGCTTTACGATGGCTGCGAAACATCATCAGACTATTGCAGAAATGTACGAGACTGATGCAGTAGATATAGAAAGAGCTGTTCAGCACTATGAACAGGCGGCCGATTACTTCAGAGGGGAAGAAAGTAATGCTTCTGCCAATAAGTGTCTTCTAAAAGTGGCTCAATATGCAGCCCAACTCGAAAATTATGAAAAAGCTGTTGGAATTTATCAAGAAGTTGCTTACGCGGCTCTGGAAAGCTCTCTTTTGAAATATAGTGCAAAGGAATACTTATTTAGAGCTGCCCTGTGTCATCTTTGTGTTGATGTACTCAATGCACAACATGCTATAGAGAGTTATATTTCAAGGTACCCCGCATTTCAAGATTCCCGTGAATACAAACTTCTGACAACCCTCATAGAAAACATCGAAGAGCAAAACGTAGATGGATACACAGAAGCCGTCAAAGACTATGATTCGATTTCTCGTCTTGATCAGTGGTATACCACAATTCTTTTACGTATTAAAAAACAAGTAAGCGAAAGCCCCGACCTACGTTAA |
| SCB_*srp54k* | ATGGTTCTCGCGGATTTAGGTCGTAAAATTACGACTGCCTTGCAGTCTTTAAGCAAGGCAACGATTATTAATGAAGAGGTTTTAAATGGAATGCTAAATGATATCTGTCGAGCACTCATCGAGGCCGACGTTAATATAAAATTAGTTAAATCTCTCAGGGAAAATGTTAAATCTGTTATTGACTTTGATGAAATGGCTGGTGGCCTCAACAAAAGGAGGATGATCCAGAGCGCAGTATTTAAGGAGCTTGTCAAATTAGTAGACCCAGGAGTCAAGCCATATCAACCCATCAAAGGCAAACCTAATGTTATTATGTTTGTTGGTTTACAAGGGTCCGGTAAAACAACCACTTGTACTAAACTAGCATATCATTATCAAAAGAAAAACTGGAAATCATGTTTGGTATGTGCAGATACATTCAGAGCTGGTGCCTATGACCAAGTCAAACAGAACTGTACAAAGGCGAGGATACCCTTTTATGGAAGTTACACAGAAGTTGATCCTGTAGTCATAGCCCAAGATGGTGTTGACATGTTTAAGAAGGAAGGTTTTGAAATCATAATTGTAGATACCAGTGGTAGACACAAACAAGAAGAATCATTGTTTGAAGAGATGTTGGCAGTTTCTAATGCTGTGAAACCAGACAATATTATTTTCGTCATGGACGCCACAATTGGTCAAGCCTGCGAGGCCCAAGCAAAAGCCTTCAAAGAAAAAGTTGATGTAGGATCTGTAATTATTACAAAATTGGATGGTCATGCAAAAGGAGGTGGTGCACTCAGTGCAGTTGCAGCCACAAACAGTCCAGTTATATTCATCGGTACAGGAGAACACATAGACGATTTAGATCCATTTAAAACAAAACCATTTATTAGTAAATTATTGGGAATGGGTGATATAGAAGGTTTAATTGATAAAGTTAACGAATTAAAGTTAGAAGATAATGAAGAATTGCTAGAAAAAATTAAACACGGACAATTTACACTCAGAGATATGTACGAACAGTTTCAAAATATAATGAAAATGGGACCTTTTTCACAAATTATGGGTATGATCCCAGGATTTAGCCAAGACTTCATGTCGAAAGGAAGCGAACAGGAATCGATGGCACGATTAAAAAGGTTGATGACAATAATGGACAGTATGAACGATTACGAATTAGACAATAGAGACGGTGCAAAATTGTTTACTAAACAAGTCGGCAGAGTGGTGAGAGTTGCGCAAGGATCCGGTGTAACAGAAAGGGAAGTAAAAGACCTCATCACGCAGTATACAAAATTCGCAGCGGTAGTAAAGAAAATGGGAGGCATCAAAGGTCTCTTCAAAGGCGGCGATATGGCTAAAAACGTTAACCACACCCAGATGGCAAAACTTAATCAACAAATGGCTAAAATGATGGACCCGAGAGTCTTGCAACAAATGGGCGGCATGGCAGGTTTGCAGAATATGATGAGACAGCTCCAGGCGGGTGCAGCTGGTGGTTTAGGGGGCTTAGGCAATCTTATGGGGGGTTTCGGTAATAAATGA |
| SCB_*dsRNase1* | TGGGCTTCGTTTGCAACCACTAGTGTGAAGTTGGCACTTAGATCTCATAGGGGGATATTGTCGTAGGAACATAGATCATATTGATTAAAAAAAACAAAATTTTTGTTTTGGCACTTAGATCACTTAGCTCGGCCCCTCTTCAAATTATTATACAAAAATAGAATCAAACAGAATTATTACTCTAACATCTTAAAGTTTAAAGATAGTCCTTATCCAAGTATTATAATACTGTTTGTCTTTCTCAGTTCAGCTATAAAGGCACCCCTAAGTATCAATTTAATATTCCCGAAACTTTTAGAAGAGGTGCATATTCAACCCTCCTTTTAAAATCAAGGCAGGTACATGCATATGAATAGCCTTTTCTTAGATTAGTTTTTTCCCAACTTAACCAATTTAGCCTATTAGATATATCATTACAAATTATATTTTTTTGAATATTCTCCTCGAATGGATTGTTTAGTCCAATTAGTACCACACATCTCTTGTTAATTGGGTTGTAAACCATTTTCCAGTATATTCCTGGAACAGGTAAGGCTTTGGTTTTTCCATTCACATACAAATATAATTCGGTTGCTTGTTGGGTCGCTTGGTGTGGTAAAGTTGCTATCCCATAAGTACCAGTCCAAATGTTTAAGTTCGCTTTTCTTCTGTCGGCATAGTTTCTAGTGCTTTGCTCAGCTTGATTCCAGTTTCCGCCATTAAAGATTTGCCACTGAGGTGCTGCATTGATAAACCTAAAAGTAGCATTTTGTTGAAATCCATAAACGAAATCCGATTTGGCTGCTAGATGTCCTCTAGAAAGGAATAAATCCCCATTACCTGTATAACTTAAGGACGAAGGTGGTAATCCCAGTAAATTATTGACAGTGAATCTTTGTCGATTCTTTGTATAGCATGTGTCAACACGATCTTTTAAATTGTAAAGGTCCCTATCTTCAAAAAACGATGGTCTGCTATTATTGACAACATTTGATTGTATTGCTGCAGTTATGTCGTAACGGGAATAAAGTGCTATTAGGGACGAAGTGTTGAAGCAGATTGTCATGGTGGATATAAACCGACCATTGTCTAAGTCAAATCCAATCTCAATAAACCTTGAATCTACTGCGCAGTTAGAATTTGTGTATCTGATCGTTGAATTAGGACTAAACCTACAGGATATGTCGGTCCAGGCATATGGCTTTTCAAAAATCTGAAATTGTTTTTTTGAAAGACACTTCGCTTCAATCGTTTTTTGGTACCTAAATCCATTGATTTCATTATTGTTTCCAGGGCATGCAAACTCAATAGTCTCTCCTTCACGAAATTTCAGTTCCTTCTCACCATAATCAGGGTACAATATTGACGAATTGCTTGGTGTTAGAACTAATGGACAATTATCATCAAACGGGTCGATCACGCAATCTGCGTTTGCAACGCTAGCCACATAATATAATATCAGAGCAAATCTGACTACCACCACACAGTTATAATACATGATTACCAGATGTTTGGAATCTTTGTGTTAAAAACCTATATTTTAATATTCATTCAGACCATGTTTATGTACGTCAACCTGTAAGAAAGTAAATTTACAGTTTAACGTTTAAACCAAATAAGATAAAGATATTATACGACCATAGAAA |
| SCB_*dsRNase2* | TGGGCTTCGTTTGCAACCACTAGTGTGAAGTTGGCACTTAGATCTCATAGGGGGATAGTGTCGTTGGAACATAGATCATATTGATTAAAAAAAACAAAATTTTTGTTTTGGCACTTAGATCACTTAGCTCGGCCCCTCTTCAAATTATTATTCAAAAATACAATTCAAATAGAATTATTACTCTAACACCTTAAAGTTTAAAGATAGTCCTTATCCAAGTATTATAAAGCTGTTTGTCTTTCTCAGTACAGCTATAAAGGCACCCCTAAGTATCAATTTAATATTCCCGAAACTTTTAGAAGAGGTGCATATTCAACCCTCCTTTTAAAATCAAGGCAGGTACATGCATATGAATAGCCTTTTCTTAGATTAGTTTTTTCCCAACTTAACCAATTTAGCCTATTAGATATATCATTACAAATTATATTTTTTTGAATATTCTCCTCGAATGGATTGTTTAGTCCAATTAGTACCACACATCTCTTGTTAATTGGGTTGTAAACCATTTTCCAGTATATTCCTGGAACAGGTAAGGCTTTGGTTTTTCCATTCACATACAAATATAATTCGGTTGCTTGTTGGGTCGCTTGGTGTGGTAAAGTTGCTATCCCATAAGTACCAGTCCAAATGTTTAAGTTCGCTTTTCTTCTGTCGGCATAGTTTCTAGTGCTTTGCTCAGCTTGATTCCAGTTTCCGCCATTAAAGATTTGCCACTGAGGTGCTGCATTGATAAACCTAAAAGTAGCATTTTGTTGAAATCCATAAACGAAATCCGATTTGGCTGCTAGATGTCCTCTAGAAAGGAATAAATCCCCATTACCTGTATAACTTAAGGACGAAGGTGGTAATCCCAGTAAATTATTGACAGTGATTCCTTGTCGTTTCTTTGTATAGCATTTGTCAACACGATCTTTTAAATTGTAAAGGTCCCTATCTTCAAAAAACGATGGTCTGCTATTATTGACAACATTTGATTGTATTGCTGCAGTTATGTCGTAACGGGAATAAAGTGCTATTAGGGACGAAGTGTTGAAGCAGATTGTCATGGTGGATATAAACCGACCATTGTCTAAGTCAAATCCAATCTCAATAAACCTTGAATCTACTGCGCAGTTAGAATTTGTGTATTTGATCGTTGTCCTAGGACTAAACCTACAGGATATATCGGTCCAGGCATATGGCTTTCCAAAAATCTGAAATTGTTTTTTTGAAAGACACTTCGCTTCAATCGTTTTTTGGTACCTAAATCCATTGATTTCATTATTGTTTCCAGGGCATGCAAACTCAATAGTCTCTCCTTTATGAAATTTCAGTTCCGTCTCACCATAATCCGGGTACAATATTGACGAATTTCTTCGTGTTAGAACTAATGGACAATCATCATCAAACGGGTCGATCACGCAATCTGCGTTTGCAACGCTAGCCACATAATATAATATCAGAGCAAATCTGACTACCACCACACAGTTATAATACATGATTACCAGATGTTTGGAATCTTTGTGTTAAAAACCTATATTTTAATATTCATTCAGACCATGTTTATGTACGTCAACCT |
| SCB_*dsRNase3* | TCAAAAATACAATTCAAATAGAATTATTACTCTAACATCTTAAAGTTTAAAGATAGTCCTTATCCAAGTATTATAATACTGTTTGTCTTTCTCAGTTCAGCTATAAAGGCACCCCTAAGTATCAATTTAATATTCCCGAAACACTTAGAAGAGGTGCATATTCAACCTTCATTTTAAAATCAAGGCAGGTACATGCATATGAATAGCCTTTTCTTAGATTAGTTTTTTCCCAACTTAACCAATTTAGCCTATTAGATATATCATTACAAATTATATTTTTTTGAATATTCTCCTCGAATGGATTGTTTAGTCCAATTAGTACCACACATCTCTTGTTAATTGGGTTGTAAACCATTTTCCAGTATATTCCTGGAACAGGTAAGGCTTTGGTTTTTCCATTCACATACAAATATAATTCGGTTGCTTGTTGGGTCGCTTGGTGTGGTAAAGTTGCTATCCCATAAGTACCAGTCCAAATGTTTAAGTTCGCTTTTCTTCTGTCGGCATAGTTTCTAGTGCTTTGCTCAGCTTGATTCCAGTTTCCGCCATTAAAGATTTGCCACTGAGGTGCTGCATTGATAAACCTAAAAGTAGCATTTTGTTGAAATCCATAAACGAAATCCGATTTGGCTGCTAGATGTCCTCTAGAAAGGAATAAATCCCCATTACCTGTATAACTTAAGGACGAAGGTGGTAATCCCAGTAAATTATTGACAGTGAATCTTTGTCGATTCTTTGTATAGCATGTGTCAACACGATCTTTTAAATTGTAAAGGTCCCTATCTTCAAAAAACGATGGTCTGCTATTATTGACAACATTTGATTGTATTGCTGCAGTTATGTCGTAACGGGAATAAAGTGCTATTAGGGACGAAGTGTTGAAGCAGATTGTCATGGTGGATATAAACCGACCATTGTCTAAGTCAAATCCAATCTCAATAAACCTTGAATCTACTGCGCAGTTAGAATTTGTGTATCTGATCGTTGAATTAGGACTAAACCTACAGGATATGTCGGTCCAGGCATATGGCTTTTCAAAAATCTGAAATTGTTTTTTTGAAAGACACTTCGCTTCAATCGTTTTTTGGTACCTAAATCCATTGATTTCATTATTGTTTCCAGGGCATGCAAACTCAATAGTCTCTCCTTCACGAAATTTCAGTTCCTTCTCACCATAATCAGGGTACAATATTGACGAATTGCTTGGTGTTAGAACTAATGGACAATTATCATCAAACGGGTCGATCACGCAATCTGCGTTTGCAACGCTAGCCACATAATATAATATCAGAGCAAATCTGACTACCACCACACAGTTATAATACATGATTACCAGATGTTTGGAATCTTTGTGTTAAAAACCTATATTTTAATATTCATTCAGACCATGTTTATGTACGTCAACCTTATAACTCATTTACACAATAAACGTTTATTTTTGAAACTGTCGTAATCAAAATCCCAGAGAACTCAATATAAACACAACACTTACCTTACATTAAATGTTAATTAGACTAATTATAGA |
| SCB_*dsRNase4* | AAATAACTTTTTTTACCCACATCTCCTCTTTTTTTTTTTTTTTTTTTTTTTTTTTTTTTTTTTTTTTTTTGTACACAATTTATTTTATTGTTACCTAATTAGTTTTATATTTGCTTAGTCAAGTTAATACAATAATCCTGTGACTTCGAGGGGCGGCAAAACATCTACTAAACTCCTAAATTTTCTGTCATTATACTCACAGGCATACGAATAACCTAATACAAGATTATCTTTATTCCAACGCCAACCTAGCCAGTTCACTTGGGATGAAACATCTCGATCACATACCATATCACTTGTCGTTACTGTTTCGTAAGGATTATTATGTCCAACGAGGATAATGGCGCTCTTACTATTGGGTTCATAAAGTAACTTCCAGAATATACCTGGAACTCTTAAACTTGGAGAATTTTTGCCACTCGTATATAAATATAATGGTTTAAGATCTCCAGTTTGTTCGTGTCGCAAAGCTGATTGTCCATATGTACCAGTCCACACTTGTAGATCTAATCTTCGATTGTGAGCATAATTCCTGGAGTCTATTTCAGCTTGATTCCAATTTCCGGCATTAAACTTGCTCCACTGAGGAGCTATGTTAATAAACCTGTAGGTAGCCTTTTGCAAAGGTTCGTATACAAAATCAGCTTTGGCAGCTAAATGTCCTTTATTAATAAAAATGTCGTTATTTATGTATCTGTTGGAACCATAATGTAGGCCTACAATTTCATTTACAGTATCTTTTCCTTCTCTAAAAATCCTGTATAGGTCCCTACTTAGCCCATAGACGCTTTCGTCTTCTATAAAATAAGGGTTTTGAAAGCCTTGGGCTCTACTATTTGTAGCAGCAGATATATTATAATGAGAATAAACTGGTATCTTATCCACCGTATCAAAACATACGTCAATATGATCGATATATCGATACGAATCTATTCGGAATCCACTTTTTAATATGACTAAATTGTGACCTCTGTTACGGCAATTAATATTGGGGTCTCCATAATTTAGTTGATATTGTCTAAATTGTCTAACAAGAGGAACGGGTTCGCTGGTACATGTGAGTGAAGAATAATCATACATTTGTCCTAGTATTTCAAAATCACCATTTTGTTTACACATTGCTGGTACTATATTGTTCATTGGTGTACCTCCAACATTGATATTACTTTCTGGACATGCAAATTCTACTTCTGTATTTGCTTCTAATCTAATATTATGTTCGCCCGGTCGAGGGTAAATAAGAGTATTGCTAATAGGATTTATGGTAAGCGGTGCAGTTGATCGCATAGGGTATATATTGCAACCAATTGAATTTCCTCTTCTTATTAAGTTACAAATACACAAGACGAAAAATAAAGTTGCCAAGTTCCTCATAATACCCAAAAGGGGTATTCAAATAAAAATTAAATTAATTATTACATAAATTTGGGATATTGAAGTTAGTTTTGAAACACACTAGAATTTTGATAATGCATCATAGTTTTCAGGACATTTTACGTTTGTAAAATGTCTACATGACACTTAAAAATTATGGGTTTG |
| SCB_*dsRNase5* | TCTTACTAAAGATTTCTTTCTAACACAGCAACAATGAGGCATTTTATGGTAATAGCAGCCTCTTTGTTAATTTCAGTAGGAGATTGTGCTCCGAGTGGGTGTGAAATAAATCCATTCGGTGGCAAAGCTCCCATGATTATAAAAAATGGTGCGAACGTACTAATAGAACCCATACCAGGTTATAGCAGACTTTCATTTCCAGTAAATACAATAGTTGAATTTGCTTGTCCTGGAACTGAAATTCAAAAGAACGGTATATCATTAGGATCACTTGTAAGTGCTACCTGTCAATCAGGTGATGTTTTCAAAATAAATGGCAACGATGTAAATTGGTCAAATTTGACCTGTCGCAACACTATTAATCCAACTGTTAAAGAAATCAGTCGAGATGGAATACTTACAAATAATCCAAATAGCGAATGTTTTGACGCCAACAAGAAATTACAGATAATTCAAATTGGATTTCAACTGGATAAGAATCGTGTCTTGGAGTCTATTAATATTTGTTTTGATACAACTACTAAACTTGCAATATATTCGTTTTATAACATATCAGCAGCTATTAATTATAGAGCTAGAAATGTTCCTAATCCTTCTTTCGCTCAAGACGATCTTTTCTATAGGATGGGTAAGTCGGTAAATACAATGTACCAACAGGTACAGAGTACGATTAATGTACTAATAGGGCTCGAACCTAGGTCACAAAAGTATGTAAACGACAGCATTTTTCCGAACCGAGGTCACTTGGCTGCCAAGGCTGATTTTATGTATGAGCCACTTCAAAGGGCCACTTATAGATACGTAAATGCAGCTCCCCAATGGAGTACTTTTAATGGAGGCAACTGGAATCAAGTAGAAACTGATGTAAGGAATTATGCTAACAACAATAAAGTGAACTTAAACATTTGGACGGGAACTTATGGACTTTCGAGCCTTCCTCACTCTCAAAATTCAAAACTTGTAAATTTGTATATTTACGTTAACAGAGAAGATAAAAAGAATTCTCCTGCGTTGCCAGTACCTAAAGTATATTGGAGATTAGTTTATGAACCCATAAAGCAACAATGTATAGTACTTGTCGGACATAATAATCCATACGAAAATATAACAACCAGTGATCAAACAATATGTGACGCAGATGTCACAGGTAACGTTAACTGGCTTAAATGGAATCAACGTGATTTGAGAACAGGATATTCTTATGCTTGTGATTGTGCCGATTATAAATTTCGAAGATTGATAACAGTTTTGCCAAAATTAACGATAAAAGGGTTATTAGTGTAAACTGTCTTAAATAATATTAATAAAAATTTAAAATAAGTAAAAAAAAAAAAAAAAAAAAAAAAAAAAGGGGGGG |
| SCB *staufenC* | ATGAAGAAACAATGTATTATTTTTCTAACCATAATTTTTCTTTCTTCAGTGACCGGAAACAAATATTCTGTGAAAGATTTACGAAATATATTTAAATATGAACTGTGTATAGAAGGTAATATTAATACCATATTTTCCAAAAAGAAGGACTCTGGGACTGAACAGAATACAAAATCTGAGGTCATTCTTAATCTCACTTCTGTGGGAACCACACAGGAATCTGAAAAGCCTGTATTATCAAGGCTAAACGAATTAGTTGGTTTTAATCAAATAGATTATTATTTCAAGTTGGAAGATGAAGAGGGACCTCCACATAACAAACTATTTACAGTGGAACTCACCTTAGGCAAGGAAAAGTATGTTGGTAAAGGGAAAAGTCTCAAAAAGGCCAAACAAGAAGCTGCTACAAAGGCCTTAGAGAAAACAGAATATGAGTTTCCCGAAATCAAAACCAAAACACCTGAAGATGTAGAAGAACTAACTCCCACAGTTCTTTTAAACAATATAGCTTCTAAATTGGGAGTTGGTGTGACATATTATCTTTTAGATCGGAATAAAGAGGAAATTCTTCATTCCAACATTATAATGACGGATAAAAAGAAATCGTATATGGAAAGACTTAATGCATCTATTTACAGTGACAAAAAATTAAGAAAGAAGAAAGATATAGATTCAACCAAGGGTCCATTTAGACTGAAATTGAAATTTGCTGATTATACATTTTTCACAACTTCACATTCCATTCAAGAGGGAAGACATGAAGTAGCTGCACAGGCCCTAGATTTTTTGGTTAAAAACAGAGATAATCTGGATATTGCTTGCTTACAAGAAGGGTCTGAGGCCGAATGCAAGAAAAACAAAGACGAACTAAAATCACCCATTTCTAAAGTATATGAAGAGGCCCAAAAAAGGAAATTAAGTATAGAATTTGAAGCAATTAAAGAGTCTGGACCATCTCATAAGAAGTCATTCAAAACAGAATGTAGACTTGGTGATATAGTAACGGAAGGTGATGGTTTTTCCAAGAAAGAATCAAAAAGGGATGCTGCTGTCAAGATGTTGCAAAAAATCTCAGAATTGGAACCACTTCCGTTAGAGGTAGAGGCTATGAATTTGAGTAAAATTGACAAGAAACAAAATAATAGGAACAAAAATAAAAAGAAAAAGCTGATCAGGAGCAAATTTGACGAACTTGGAATGATGCTTGACAAAGTGGGAAAATCTATTGAACGAATAGCTAGCAATGTTTTTGGGAAAGAAGATCAGCCTACAGATTCCGTTGAAGACGTGGAGAGTAACGAAGAAATAAAATCTGAGAAGAAAAAAGATAAACAACAAAGACCTCACACTAAAAAATCATCATTCCAAGATGAACTGCTAGAATTAAGCAACATTTTGGGATTTAAAGTGTCATATACAGATTTTTCAGAAAAAGACACCCATGCGTCTTTACTTTCCCTTTACACCAATCCAGAATATATTTGTTTTGGTGACGGTAAAACCGAGTCCAATGCTAGAAATAATGCTGCAAATGAAGGTCTGAAGCTTCTGGAAAAAATGGGTATTTATGATTTATTTCAAGAGCAGAAGGAGACTGATTTGGAGAGAGATACCAAAGAAGGAGTGAGAATAGCTATGCAGCATATAGTTACAAAAAAGAAAGAAGAATTGTGA |
|  | **CPB gene sequences** |
| **Gene** | **Gene sequence** |
| CPB_*ef1α* | CCCACAGTTAACTGGGATGAAGAAAGATACAACGAGTGCAAAGACAAAATTTTGCCGTACCTTAGGAAATTAGGATTCAACTATAATAAAGATTTATTCTTTTTACCTTGTTCTGGGCAAACAGGGCAAGGTTTGAAAGATAAAGTAGATGAAAAAATTTGCCCCTGGTACAGGGGGGATGCCTTTATTCCCTTTATTGATAATATCCCATCGCTCAATCGCAAAGCAGACGGACCATTTATAATGCCAATTGTAGACAAATATAAAGATATGGGAACTGTGCTGATGGGCAAGGTTGAAT |
| CPB_*l8e* | ACTTTGCACTTTCATTTAAGGAAATGTTTTGCCTGTTGGAACCATGCCAGAAGGTACAATCGTATGTAACTTAGAAGAAAAGACTGGCGATCGTGGTCGTCTTGCAAGGGCATCTGGAAACTATGCAACAGTTATTGCCCACAATCATGACACAAAAAAAACCAGGGTCAAGTTACCATCTGGTGCCAAAAAAGTCATTCCTTCCAACAATAGGGCCATGGTTGGTATTGTTGCAGGGGGTGGTCGTATCGACAAACCAATTCTTAAAGCTGGACGTGCCTATCACAAATACAAGGTTAAGCGTAACTGCTGGCCCAAGGTACGTGGTGTTGCTATGAATCCCGTCGAACATCCCCATGGTGGTGGTAACCATCAACACATTGGTAAAGCATCCACTGTTAAAAGAGGTACCTCTGCTGGTCGTAAGGTTGGTCTCATTGCCGCCAGGAGAACCGGTCGTATTCGGGGTGGTAAAGTGGATGCCAAGAAGGAAGATTAAATTTAAATTGTTAATGCTTGATAATATGTCAATATGTTTTTTCTGGATCGTCTGTGAAGTAAAACTTGTGTTTCATTAGAAATATATTGACTTTGATAATTGAAA |
| CPB_*β-actin* | GCACGAGGTTTTTCTGTCTAGTGAGCAGTGTCCAACCTCAAAAGACAACATGTGTGACGACGATGTAGCGGCTCTTGTCGTAGACAATGGATCCGGTATGTGCAAAGCCGGTTTCGCAGGAGATGACGCACCCCGTGCCGTCTTCCCCTCGATCGTCGGTCGCCCAAGGCATCAAGGAGTCATGGTCGGTATGGGACAAAAGGACTCATACGTAGGAGATGAAGCCCAAAGCAAAAGAGGTATCCTCACCCTGAAATACCCCATCGAACACGGTATCATCACCAACTGGGATGACATGGAAAAGATCTGGCACCACACCTTCTACAACGAACTCCGTGTCGCTCCAGAAGAACACCCAGTCCTTCTCACTGAAGCTCCACTCAACCCCAAAGCCAACAGGGAGAAGATGACCCAAATCATGTTTGAGACCTTCAACACACCCGCCATGTATGTAGCCATCCAAGCTGTACTCTCCTTGTACGCTTCTGGTCGTACCACCGGTATCGTCTTGGACTCTGGAGATGGTGTCACCCACACCGTACCAATCTACGAAGGTTACGCTCTTCCCCACGCCATCCTCCGTTTGGACTTGGCTGGACGTGACTTGACCGACTACCTCATGAAAATCCTCACCGAGAGGGGTTACTCATTCACCACCACAGCTGAAAGGGAAATTGTCCGTGACATCAAGGAGAAACTTTGCTATGTTGCCCTCGACTTCGAACAGGAAATGGCCACCGCTGCTGCTTCAACCTCCCTCGAAAAGAGCTACGAACTTCCCGATGGACAGGTCATCACCATTGGTAACGAGAGGTTCCGTTGCCCAGAAGCCTTGTTCCAACCTTCCTTCTTGGGTATGGAATCTAGCGGTATCCACGAAACCGTCTACAACTCCATCATGAAGTGTGACGTTGATATCCGTAAGGACTTGTACGCCAACACCGTCCTCTCTGGAGGTACCACCATGTACCCTGGTATTGCTGACCGTATGCAGAAGGAAATCACCGCTCTTGCCCCATCCACCATCAAGATCAAGATCATCGCTCCCCCAGAAAGGAAATACTCCGTATGGATCGGTGGATCCATCTTGGCTTCCCTGTCCACCTTCCAACAGATGTGGATCTCCAAACAAGAATACGACGAATCCGGCCCTGGAATCGTTCACCGCAAGTGCTTCTAAGCAATTTAATTGCATTTTACCTAATAAGCTACCTTATATCATGTTGTTACTACTGTATTTTACATCAACAGTTGAATTACATTGCGACCGTGGTTGTTACTGCAAGACAGACTCTTAACATCGATTAACATC |
| CPB *staufenC* | ATGTTGGAATTAACGATTCATTTCAATGTGTTACTTGTTTTTTTGGTTGCTTTTTGTGGAGGGTATTCGGAAGAAGGAATAAGGCAAGCATTTGGAGCAAAATCATGTATTCAAGCATCACTTTCATCACTAGATCTTCATTCTTTCGGAGTAAAAGAGTCACAAAACTCCATTATAGGGAATATAAAGTCTGCAAGAGGAGCAGAAAATACTGACAAATCATCTTTGTCAAGACTGAATGAGCTAGTACAGTTTAATGAGATTGACTATTATTTCAAGCTAGAAAAAGAGGAAGGCCTGCCTCACGATAAAGTCTTTACAGTGTCCCTTACACTGGGAACAGAAACATATATAGGTGAAGGTAAAAGTCTGAAAAAGGCCAAGCAAAATGTAGCAGCTATAGCGTTACATGAGACGAGGTATGAAACACCCCCTGTGAAGGAACCATCAGAAAATGAAGAGTCATTAACTCCTACGGTAATGTTAAATAATCTTGGGGCAAAACTTGGAATAGGGATCACTTACTATTTAATTGACAAGGAGAAGCAGCATATACTCAACTCCAACCTAGTTGTTAGTGAAAATTCCAAAAAATCCTATGTTCAAAAACTGAATGACTCCATTTATTCGAATAAAAACCTACGAATGAAAAAAGATATTGAAAATACCAAAGGACCTTTTAAAATAAAGGTTCAAGTAGGAGATCAAGTTTTTTCTGGCTATGCCCATTCAATACAAGCAGCAAGACATCAGGCTGCATCTAATGCCTTGGATTTCTTGATAAAAAATAAGGATAGTCTTGATCTGGATTGTCTTAAAGAAGGTTCAGAAGAGCAATGCAAAAAAGCCAAACAAAACTTGAAGTCGCCAGTATCTTTAGTTTATGAGTCAGCACAGATGCGAAAACTTGATGTAGAGTTTGAAATAATTAAAGAATTTGGTCCTCCTCACAAGAAAACATTCGTTACAGAATGTAGAGTGGGTCATTTGACAACAACAGGAGAGGGAAGGTCAAAAAAGGCATCTAAGAAAGCTGCTGCTGAAGACATGTTAGAGAAAATGTCAGAGTTAGAACCGATACCTCAAGAAGTGCAAGTTAAAAGTATGCTTAAAGATAAAAAGAAGAAAAATAAGAAGAAGAAGATCATTAAAAATAAGTTGGATGAAATTAGCATGACTGTTGGAAATGTGATAGACTCTGTGGTGGGATTTGGCAAAGACATTCTGGCTGATAAGAAGGGTGATGACAAAACCGACAAAAATTCGTCAGACGGTCCTAAATCCAAGAAAAGCAAGAAGTCGGAAAATTTAAAGCAAACCTATCAAGACCAACTTCTGGAAATGAGTAATGCTCTGAATTTTGAAA;TATCTTACGCAGATTTTGAAGAAGGAAGTAAACATTTTTCCCTCCTCTCGTTGCACATCAATCCTGAATATTTGTGTTTTGGAGAGGGATCCAATAAAATGCAATCGCGAAATAAAGCTGCAGATAAGGGTTTGGATTTATTGGGCAAAATGGGTTTATTTGATATTTTGAATGATCAGAAAACTGTTCCTTTGGAAAGGGATACCAAAGAAGCAGTACATCATGTTCTGGAACATCACATATCTCAAGATAAAGATGAACTGTAA |
|  | **Control dsRNA sequences** |
| **Gene** | **Sequence** |
| *gfp* | ATGAGTAAAGGAGAAGAACTTTTCACTGGAGTTGTCCCAATTCTTGTTGAATTAGATGGTGATGTTAATGGGCACAAATTTTCTGTCAGTGGAGAGGGTGAAGGTGATGCAACATACGGAAAACTTACCCTTAAATTTATTTGCACTACTGGAAAACTACCTGTTCCATGGGTAAGTTTAAACATATATATACTAACTAACCCTGATTATTTAAATTTTCAGCCAACACTTGTCACTACTTTCTGTTATGGTGTTCAATGCTTCTCGAGATACCCAGATCATATGAAACGGCATGACTTTTTCAAGAGTGCCATGCCCGAAGGTTATGTACAGGAAAGAACTATATTTTTCAAAGATGACGGGAACTACAAGACACGTAAGTTTAAACAGTTCGGTACTAACTAACCATACATATTTAAATTTTCAGGTGCTGAAGTCAAGTTTGAAGGTGATACCCTTGTTAATAGAATCGAGTTAAAAGGTATTGATTTTAAAGAAGATGGAAACATTCTTGGACACAAATTGGAATACAACTATAACTCACACAATGTATACATCATGGCAGACAAACAAAAGAATGGAATCAAAGTTGTAAGTTTAAACATGATTTTACTAACTAACTAATCTGATTTAAATTTTCAGAACTTCAAAATTAGACACAACATTGAAGATGGAAGCATTCAACTAGCAGACCATTATCAACAAAATACTCCAATTGGCGATGGCCCTGTCCTTTTACCAGACAACCATTACCTGTCCACACAATCTGCCCTTTCGAAAGATCCCAACGAAAAGAGAGACCACATGGTCCTTCTTGAGTTTGTAACAGCTGCTGGGAATACACATGGCATGGATGAG |
| dsRNA sequence from empty pl4440 vector | TAATACGACTCACTATAGGGAGACCGGCAGATCTGATATCATCGATGAATTCGAGCTCCACCGCGGTGGCGGCCGCTCTAGAACTAGTGGATCCACCGGTTCCATGGCTAGCCACGTGACGCGTGGATCCCCCGGGCTGCAGGAATTCGATATCAAGCTTATCGATACCGTCGACCTCGAGGGGGGGCCCGGTACCCAATTCGCCCTATAGTGAGTCGTATTA |

**Figure S1. *dsRNase* gene expression in insects injected with water or *gfp* dsRNA.** Normalized transcript level was set to “1.0” in water-injected SCB, and relative transcript levels in *gfp* dsRNA-injected SCB were calculated. Data are mean relative quantity ±SEM, n=3. No significant changes in the transcript levels were found in *t*-tests (*P*≥0.05).

**A**

**SCB CPB**

**
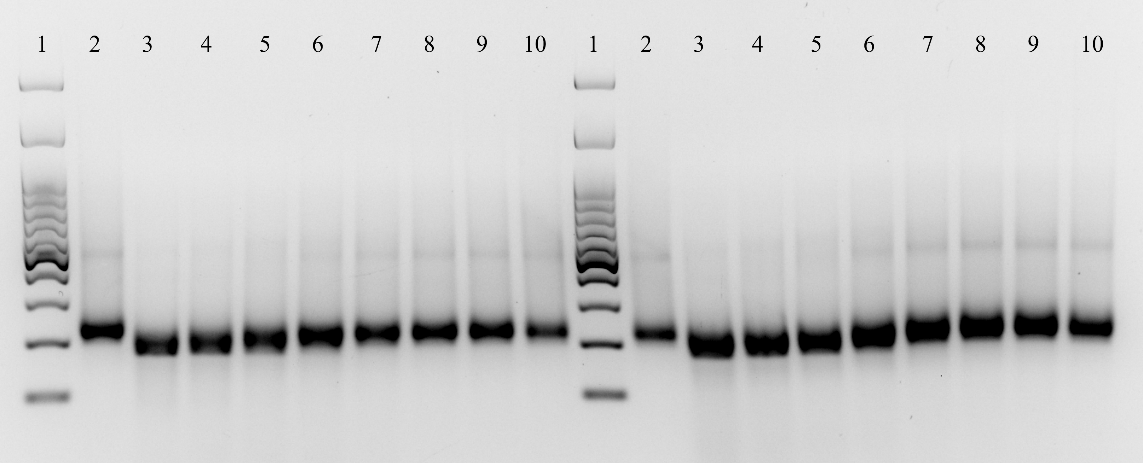
**

**SCB CPB**

**B**

**
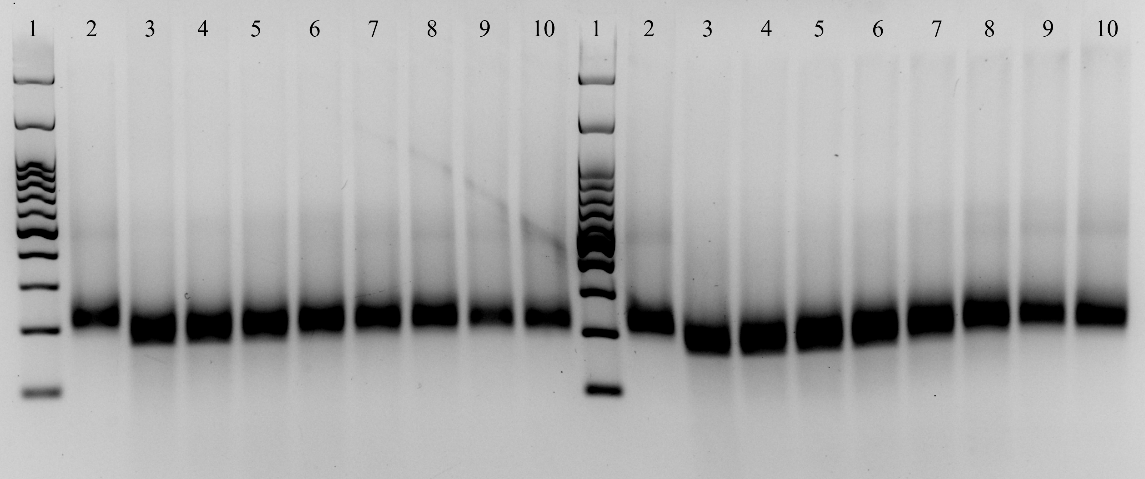
**

**Figure S2. Elimination of dsRNA degradation in SCB and CPB digestive fluids. A)** Samples were heated to 100°C prior to dsRNA incubation. **B)** 100 mM EDTA was added to the reaction mixtures. 1= 100bp DNA ladder; 2= 0% (control) digestive fluid; 3= 5.00%; 4= 2.50%; 5= 1.25%; 6= 0.625%; 7 = 0.312%; 8= 0.156%; 9= 0.078% and 10 = 0.039% of digestive fluid.

**Figure S3: *staufenC* gene transcript levels in whole bodies of SCB and CPB.** Normalized transcript levels were set to “1.0” in CPB, and relative transcript levels in SCB were calculated. Data are mean relative quantity ±SEM. No significant differences between the two insects were found in *t*-tests (*P*≥0.05), n=3.

**Figure S4. *staufenC* gene transcript levels in different tissues of SCB.** Normalized expression was set to “1” in the gut, and relative fold change of each gene was calculated in other tissues. Bars represent mean relative quantity ±SEM, n=3. Letters above bars denote significant differences between tissues. Means with the same letter are not significantly different (*P*>0.05) according to Tukey’s HSD tests (one-way ANOVA).
